# The relevance of late MSA mandibles on the emergence of modern morphology in Northern Africa

**DOI:** 10.1038/s41598-022-12607-5

**Published:** 2022-05-25

**Authors:** Inga Bergmann, Jean-Jacques Hublin, Abdelouahed Ben-Ncer, Fatima Zohra Sbihi-Alaoui, Philipp Gunz, Sarah E. Freidline

**Affiliations:** 1 Department of Human Evolution, Max Planck Institute for Evolutionary Anthropology, Deutscher Platz 6, 04103 Leipzig, Germany; 2 Chaire de Paléoanthropologie, Collège de France, 11, place Marcellin Berthelot, 75005 Paris, France; 3 Institut National des Sciences de l’Archéologie et du Patrimoine, Hay Riad 5, 10000 Rabat, Morocco; 4 Department of Anthropology, University of Central Florida, 4000 Central Florida Blvd., Orlando, USA

**Keywords:** Evolution, Anatomy

## Abstract

North Africa is a key area for understanding hominin population movements and the expansion of our species. It is home to the earliest currently known *Homo sapiens* (Jebel Irhoud) and several late Middle Stone Age (MSA) fossils, notably Kébibat, Contrebandiers 1, Dar-es-Soltane II H5 and El Harhoura. Mostly referred to as “Aterian” they fill a gap in the North African fossil record between Jebel Irhoud and Iberomaurusians. We explore morphological continuity in this region by quantifying mandibular shape using 3D (semi)landmark geometric morphometric methods in a comparative framework of late Early and Middle Pleistocene hominins (n = 15), Neanderthals (n = 27) and *H. sapiens* (n = 145). We discovered a set of mixed features among late MSA fossils that is in line with an accretion of modern traits through time and an ongoing masticatory gracilization process. In Northern Africa, Aterians display similarities to Iberomaurusians and recent humans in the area as well as to the Tighenif and Thomas Quarry hominins, suggesting a greater time depth for regional continuity than previously assumed. The evidence we lay out for a long-term succession of hominins and humans emphasizes North Africa’s role as source area of the earliest *H. sapiens*.

## Introduction

For most of the Plio-Pleistocene Africa has been the core continent of hominin evolution. Genetic and fossil evidence suggest that the ancestry of all people living today can be traced back to Africa^1–3^, but little is known about its population dynamics. Situated at a shifting interface between Palaearctic and Afrotropical ecozones, the northern part of the continent played a critical part in *H. sapien's* expansion and represents a strategic area for encounters between Middle Pleistocene hominins. The redating of the Kabwe^4^ and Jebel Irhoud fossils to around 300 ka BP^5^ questions the direct descent of our lineage from a group of African large-brained Middle Pleistocene specimens either classified as *H. rhodesiensis* or *Homo heidelbergensis* s.l., namely Kabwe (Broken Hill)*,* Bodo, and Hopefield^6,7^. With the evidence from Jebel Irhoud^3^, North African late Early/Middle Pleistocene hominins from Tighenif or Thomas Quarry became potential candidates for the ancestral morphology of Neanderthals and *H. sapiens*^7,8^. Yet, regional continuity within Africa has been hotly debated given a sparse fossil record and complex environmental dynamics^9,10^*.*

**Figure 1:**
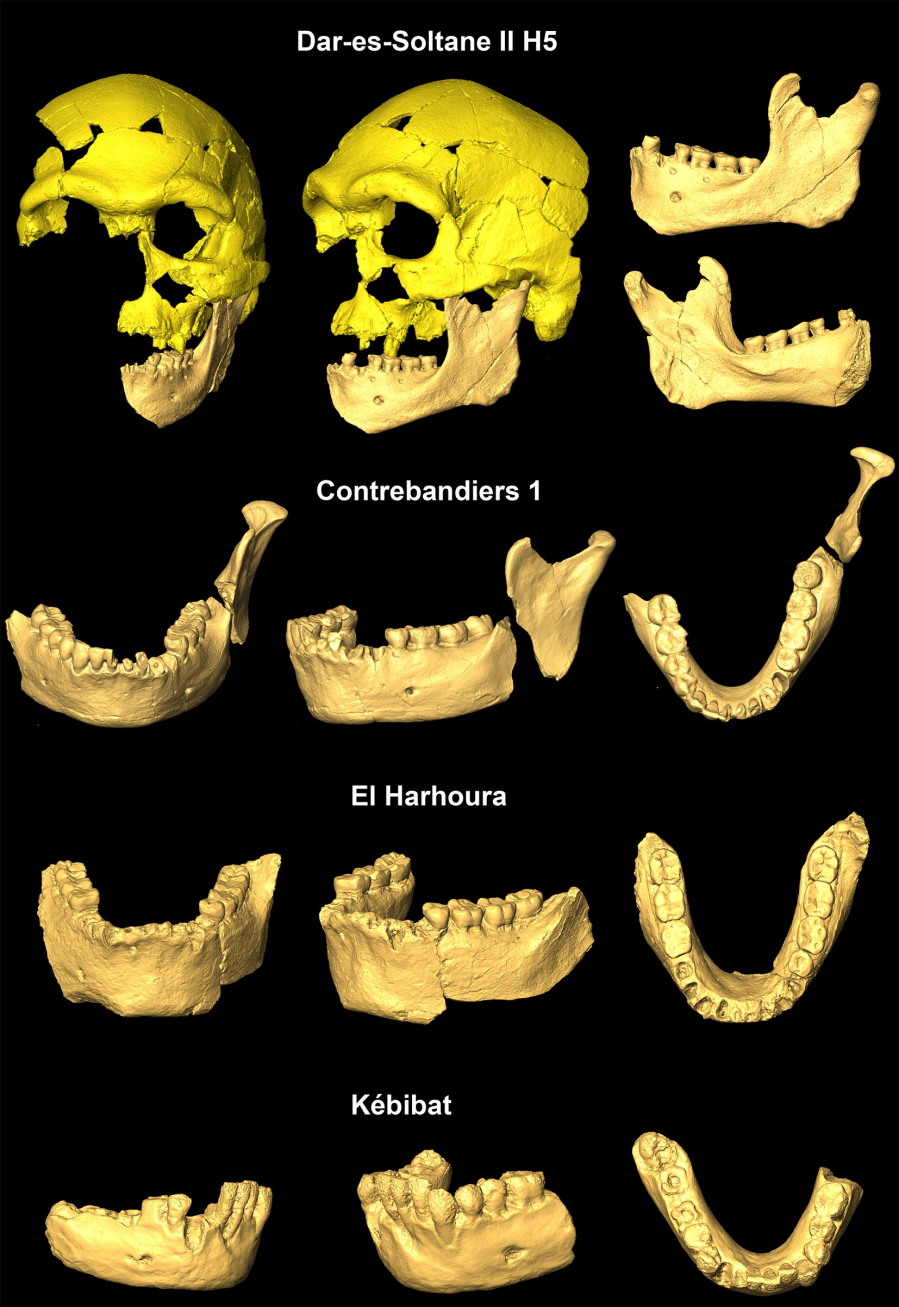
Kébibat and Aterian mandibular specimens.

The aim of our study is to “fill a gap” between the Jebel Irhoud and Iberomaurusian humans with the first detailed morphometric analysis of four Moroccan mandibles dated to MIS 6-4 (Fig. 1). As mandibular remains carry phylogenetic signals^11^ and are numerous in paleoanthropological contexts, these specimens give us insight into people living at the time of the last out-of-Africa dispersal^12,13^. Three of the studied specimens were associated with the “Aterian”^14^, a typological variant of the Maghrebian MSA lithic industries that dominates the archaeological record west of the Nile Valley between 145^15^ and 30 ka BP. As early as 142 ka BP, Aterian material culture documents the emergence of modern behavior^14,16^ in the form of perforated and ochre-decorated *Tricia (Nassarius)* shells^17^, hearths and stone-walled structures^18^ as well as formal bone tools^19–21^. The three hereafter referred to as Aterian individuals (Supplementary Table S1) were recovered in close vicinity to each other from cave sites in littoral Maghreb, namely Grotte des Contrebandiers, Dar-es-Soltane II and El Harhoura 1. Likewise of late MSA origin, the fossil mandible from Kébibat will be referred to without group affiliation as it lacks any archaeological information.

While early *H. sapiens* from South Africa display a rather large variation^22,23^, the Jebel Irhoud and Aterian humans share a robust craniofacial/mandibular morphology with late Early/Middle Pleistocene hominins from North Africa^24–26^, in particular a small number of archaic features. Broad braincases and faces with well defined superstructures in the Jebel Irhoud, Contrebandiers and Dar-es-Soltane II 5 crania were reported as intermediate between preceding (Tighenif, Kébibat) and subsequent groups (Iberomau-rusians)^24,26^. A similar pattern of progressive evolution was proposed for the associated mandibles (Tighenif, Thomas Quarry, Sidi Abderrhaman, Kébibat, Jebel Irhoud, Contrebandiers)^27^, but challenged by the presence of a modern-looking face and high cranial capacity in the Jebel Irhoud humans^3^ that contrast the geologically younger but more archaic morphology of Kébibat^27^ (140 ka) and the smaller cranial capacity of the older Salé cranium (400 ka)^28^. As indicated above, the massive Iberomaurusian crania have been sometimes compared to Aterian and Jebel Irhoud skulls from the same area^24,26^. However, the apparent archaeological hiatus at the Middle/Later Stone Age transition^29,30^ renders population continuity between Aterians and Iberomaurusians an unresolved case.

### Geological context of late MSA sites in Morocco and description of associated fossils

In 1933, M. Allenda, who supervised communal works in Kébibat, a suburb of Rabat, discovered a fossil mandible in a pile of cobbles intended for roadworks^31^. Thereupon he located the reburied remains of this adolescent individual (15–16 yrs) in the coastal quarry Mifsud-Giudice, consisting of 23 cranial fragments and an incomplete left maxilla, heavily damaged by a detonation and without any associated archaeological material. The geologic provenance of this so-called “Rabat Man” could be tracked via an imprint of the palate and its molars in the calcarenite limestone. The associated sedimentary unit 2 from sequence 2 was dated by Infrared Stimulated Luminescence (IRSL) to 137 ± 7 ka BP^32^, which is in line with previous dating attempts of an underlying marine shell layer^33^. The Kébibat human remains (Fig. 1) have been attributed to a population featuring a mixture of archaic and modern traits^8,27,28^, in accord with the mosaic character of human morphology at that time^11^.

After J. Roche had conducted some test excavations in the Smuggler's Cave (Grotte des Contrebandiers) between 1955 and 1957 a human mandible (Fig. 1)—hereafter referred to as Contrebandiers 1—was recovered in layer 9, OSL-dated to 111–92 ka BP^34^. This layer corresponds to the lower part of layer 4 in the central area of recent excavations^35^ and exposed another human calvaria just before the end of Roche’s excavations in 1975. The resumption of Roche’s excavations has been directed by M. A. El Hajraoui and H. Dibble (deceased 2018) since 2006^18^. Due to its high robusticity in bone and dentition Contrebandiers 1, consisting of an incomplete corpus and three ramus fragments^18,36^, was initially thought to originate from an Acheulian deposit^37^. Accordingly, it displays an inferior transverse torus, a prognathic and vertical symphysis as well as a mental foramen situated under P_4/_M_1_. In contrast, the anteroposterior decreasing corpus height represents a *H. sapiens* characteristic.

Near Rabat, a partial cranium with the left half of the face (H5), an adolescent mandibular corpus (H4) and an infant calvaria (H3) were excavated by A. Debénath in 1975 in the cave of Dar-es-Soltane II^24^. The specimens came from an archaeologically sterile marine sand deposit (unit 7), immediately overlain by an Aterian MSA (Middle Stone Age) layer^38^. Based on OSL, an age possibly beyond 100 ka was proposed for Unit 7^39^. Amino acid racemization on *Patella* shells calibrated by C14/AMS rather point to a maximum age of 85–75 ka BP^40^. The morphology of individual H5 has been interpreted as intermediate between Jebel Irhoud and Iberomaurusians^24,41^, exhibiting extraordinary breadth dimensions, marked brow ridges and a high robusticity. Some facial structures were even described as Neanderthal-like. Yet, the associated left hemimandible, hereafter referred to as Dar-es-Soltane II 5, lacks a retromolar gap and its large mental foramen is under P4 (Fig. 1). It belongs to a mature individual and is one of the largest specimens studied. The ramus is wide with an expanded gonial area showing medial pterygoid tubercles, but also a marked masseteric fossa. Despite its unpreserved inferior symphysis, a curved mental profile is discernible.

In the second archaeological layer (see Fig. 7 in^18^) of the Zouhrah Cave (El Harhoura 1), a large and robust mandibular corpus (Fig. 1) and a large isolated canine were unearthed during a salvage excavation in 1977/1978^25,42^. Burnt sandstone structures in the underlying layer 1 date to 32,150 ± 4800 BP by ƴ TL^43^. The relatively young C14 date of 25,580 ± 130 BP on *Helix* shells^44^ from layer 1 or 2 has been suspected to result from secondary colonization by these gastropods^40^. Unpublished Uranium–Thorium dates of a spelothem that caps layer 2 rather indicate that the El Harhoura mandible has a minimum age of approximately 66 ka BP. Despite such relatively recent age and the presence of a human chin, it displays a uniform corpus height, a prognathic and U-shaped dental arcade as well as an inferior transverse torus.

### Information on North African late Early/Middle Pleistocene sites in the current study

**Figure 2:**
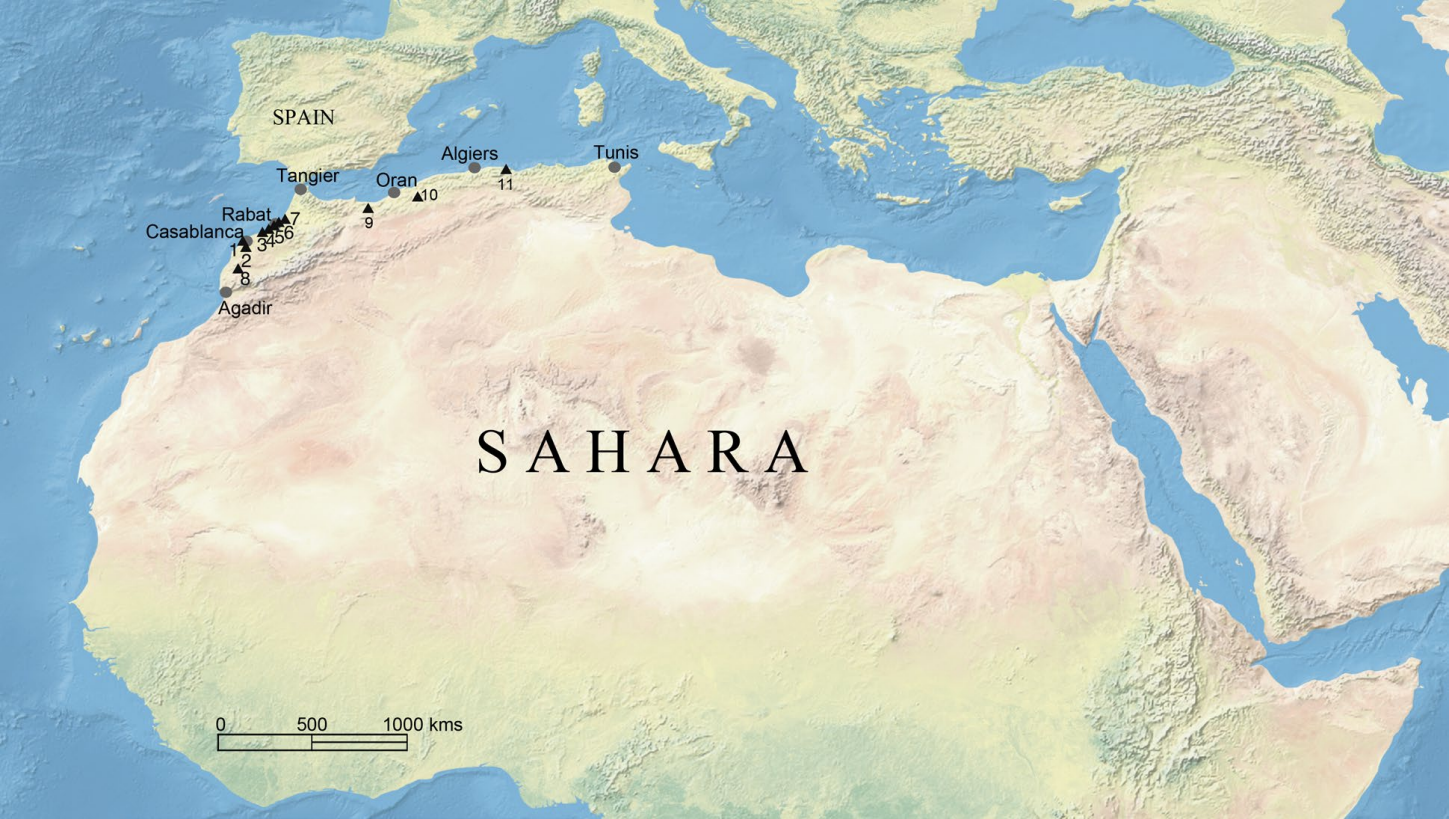
Map with North African sites mentioned in the text. *1* Sidi Abderrahman, *2* Thomas Quarries, *3* Grotte des Contrebandiers, *4* El Harhoura II, *5* Dar-es-Soltane, *6* Kébibat, *7* Salé, *8* Jebel Irhoud, *9 *Taforalt, *10* Tighenif, *11* Afalou Bou Rhummel. Topographic map taken from https://www.naturalearthdata.com/ and edited in PDF-XChange Editor v. 8 (PDF-XChange Co. Ltd, Bolney).

Between 1969 and 2008, the sandstone quarries of Thomas I and III near Casablanca (Fig. 2) yielded several craniofacial and dental remains of fossil hominins associated with Acheulian lithics and abundant fauna^45^. Thomas I yielded an eponymous left corpus fragment^46,47^, a complete jaw (hereafter referred to as Thomas Gh10717) and a juvenile right corpus fragment. Geochronology, biostratigraphy and ESR dating of tooth enamel point towards an early Middle Pleistocene age of around 700–600 ka BP^45,48–50^.

From 1954 to 1956 the Tighenif quarry east of Mascara in Algeria (Fig. 2), previously known as Ternifine or Palikao^51^, yielded two nearly complete jaws (Tighenif 1 + 3) and a hemimandible (Tighenif 2). Geological and micromammalian evidence indicate a short site formation process characterized by a seasonal lake in an open environment^52^. Biostratigraphy and paleomagnetism initially placed the site close to the lower Middle Pleistocene boundary around 700 ka BP, its lithic assemblage was assigned to the late Lower Acheulean. After biostratigraphic revision, a late Early Pleistocene age within the Jaramillo Event around 1 Ma has been suggested^53^. This seems to be confirmed by a reevaluation of the faunal association^54–56^. For data analysis, we group the Tighenif fossils with the African Middle Pleistocene.

## Objectives and working hypotheses

Not all of the early *H. sapiens* mandibles fall into the morphological range of recent modern humans^3,11^, making it seem likely that the emergence of diagnostic *H. sapiens* features follows an accretional pattern. If modern mandibular morphology emerged by such a shift in the frequency of modern traits through time, we expect a mix between archaic and modern traits in all four late MSA mandibles from Morocco (Fig. 1). Aterian remains have been described as less archaic than the earliest currently known *H. sapiens* from Jebel Irhoud, but not as modern as Iberomaurusian or European Upper Paleolithic groups^23,24,41^. In this regard, Aterian mandibular shape and size might fill a gap in the human fossil record that currently exists between early and later *H. sapiens*^3,11^. The isolated Kébibat hominin has been classified as a morphological link between archaic and modern populations in Africa^8^. The occurence of this non-modern specimen well after the emergence of the first *H. sapiens*^3,5^, reflects a non-linear evolution towards *H. sapiens* and may indicate rapid shifts in the frequency of certain traits.

Apart from the Jebel Irhoud and Aterian humans, the Maghreb provided fossils of late Early/Middle Pleistocene hominins (Fig. 2), offering unique insights into early phases of *H. sapiens* evolution. Genetic evidence suggests regional continuity in the Maghreb since the Epipaleolithic^57,58^, but biological exchanges with the Eastern Mediterranean Basin and the Sahelian zone to the south are documented as well^57,59^.Craniofacial studies question a progressive evolution towards *H.* *sapiens*^3,28,41^, but disclose notable similarities between Middle and Late Pleistocene populations in the area^24–27^. In order to track the evolution of Northern African populations and potential influences from adjacent areas during the Middle and Late Pleistocene, we quantify mandibular variation in Kébibat, Contrebandiers 1, Dar-es-Soltane II 5 and El Harhoura along with late Early/Middle Pleistocene hominins, Neanderthals, early and later *H. sapiens* (Supplementary S1, S2, Figs. S1 and S2, Table S1).

We use 3D geometric morphometrics to perform principal component analyses in Procrustes shape space and to visualize group mean shapes as well as differences in absolute mandibular dimensions. The strength of this approach is the perspective on mandibular shape separately from size, allowing us to track shape continuity across different time periods. The complementary record of discrete mandibular traits provides insights into differences between individuals.

## Results and discussion

**Figure 3:**
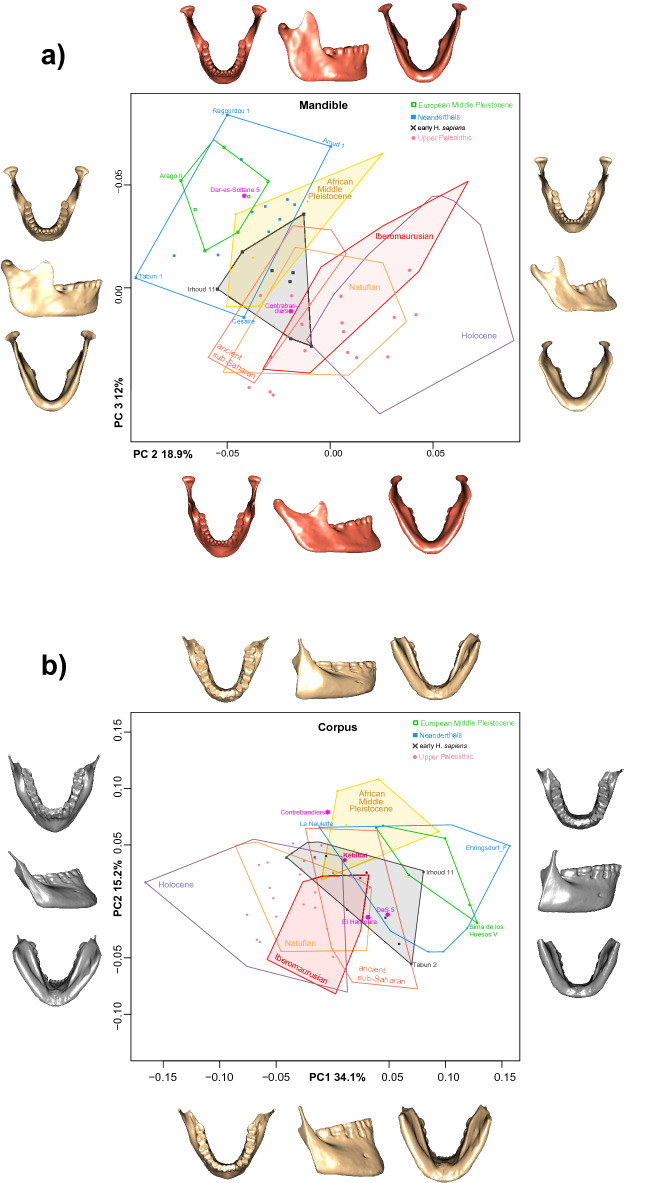
Principal component (PC) analyses of the pooled samples of the mandible (*n* = 166) and corpus (n = 192) data sets in shape space. Warps of PC extremes are displayed in grey (PC1), in bone color (PC2) and red (PC3) for ± 3 SD. They illustrate shape changes that drive mandibular variation along each PC.

**Figure 4:**
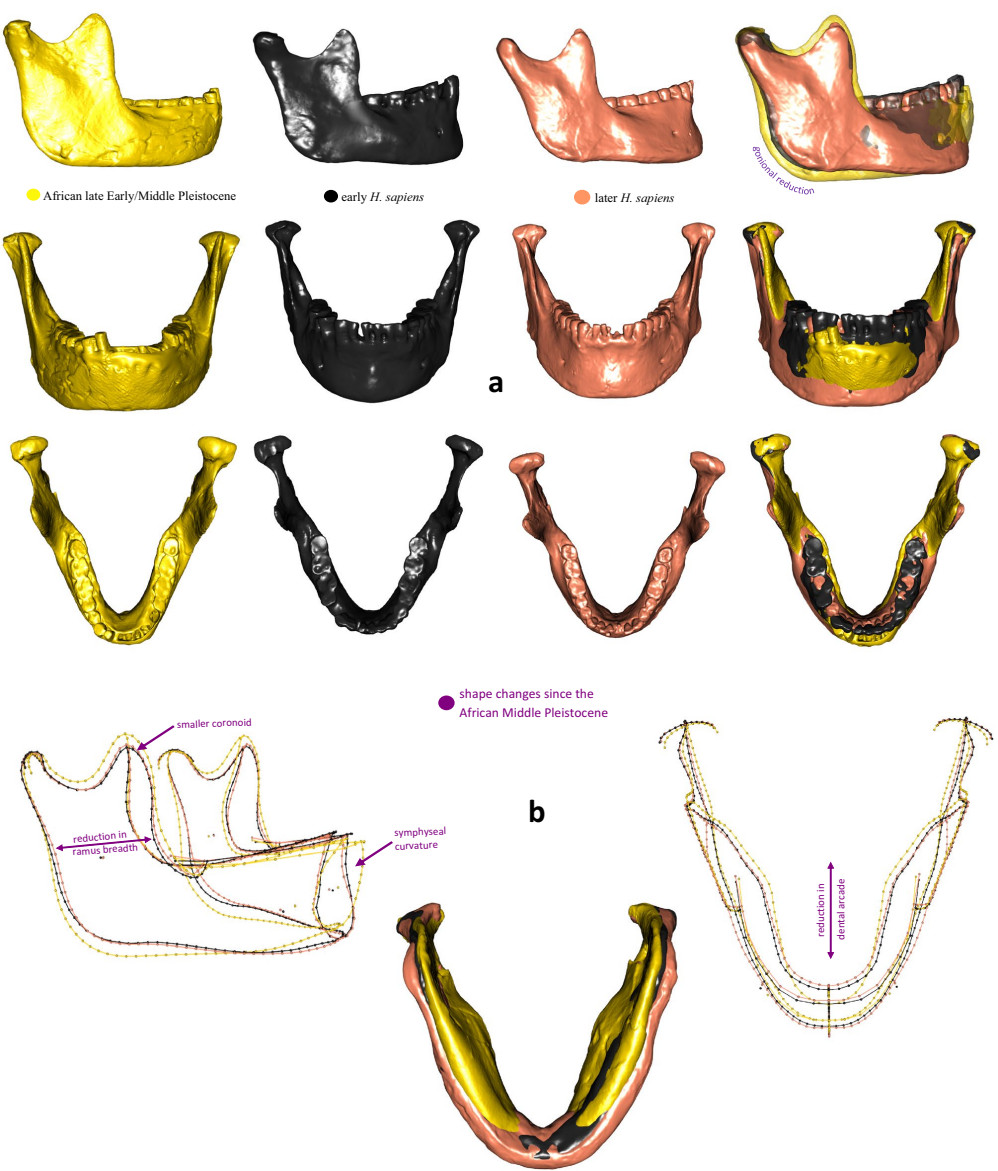
Mean shapes of African late Early/Middle Pleistocene hominins, early *Homo sapiens*, and later *H. sapiens* (Iberomaurusians/Natufians/ancient sub-Saharans/Upper Paleolithic/Holocene) warped onto a 3D surface model of an individual from the respective group (**a**) and as wireframes (**b**).

**Figure 5:**
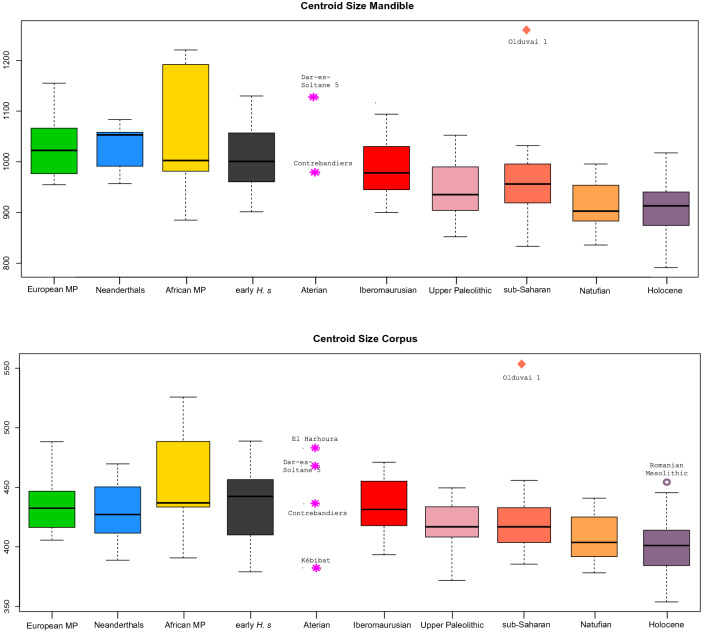
Box plots depicting centroid size for each group in each data set. Horizontal lines represent the median of each group. Boxes show the interquartile range (IQR, 25th to 75th percentile). Whiskers extend to 1.5 times IQR, but minimum to the lower/upper 25% of the data.

In the complete mandible data set Principal Component (PC) 1 accounts for 29.7% of the total variation and reveals substantial overlap between groups (Supplementary Fig. S3). Along PC 2, accounting for 18.9% of total variation, archaic groups (late Early/Middle Pleistocene hominins and Neanderthals) separate well from most *H. sapiens*, however, early *H. sapiens* and ancient sub-Saharans overlap with both clusters (Fig. 3a). The Tighenif mandibles are distinct from Neanderthals. European Middle Pleistocene individuals and Neanderthals fall towards the negative end of PC 2 while Holocene humans and Iberomaurusians plot towards the positive extreme. Natufian and Upper Paleolithic specimens attain intermediate scores, but intersect considerably the former two groups. In the corpus data set archaic groups separate best from *H. sapiens* along PC1 (Fig. 3b), accounting for 34.1% of the total variation. Again, early *H. sapiens* and ancient sub-Saharans fall in the middle. A three-dimensional visualization of group mean shapes (Fig. 4) supports the intermediate position of early *H. sapiens* disclosing a morphological succession in time from African Middle Pleistocene hominins to later *H. sapiens*. Except for Jebel Irhoud 11^3^ (Table 1; Supplementary Tables S3–S7), early *H. sapiens* display a reduction in dental arcade length, gonial area, ramus breadth and coronoid size, pushing back the onset of masticatory gracilization^11^ to the Middle/Late Pleistocene transition. This process persists into the Holocene, explaining why Holocene people feature on average the smallest mandibles followed by Natufian, ancient sub-Saharan and Upper Paleolithic groups (Fig. 5; Supplementary Tables S3–S7). Iberomaurusians are clearly larger than their contemporaries, covering a similar size range as Aterians and early *H. sapiens*. Out of all samples, African Middle Pleistocene mandibles show on average the largest dimensions.

**Table 1:** Archaic discrete traits of North African late Early/Middle Pleistocene specimens, Irhoud 11, Kébibat, and Aterians.

	North African MP	Irhoud 11	Kébibat	Contrebandiers 1	Dar-es-Soltane II 5	El Harhoura
Inferior transverse torus	✓	✓	✓	✓	?	✓
Archaic symphysis	✓	✓	✓	✓	–	–
Uniform corpus height	✓	–	?	–	–	✓
Prognathism	✓	✓	?	✓	–	✓
Wide ramus	✓	✓	?	–	✓	?
Large gonion	✓	✓	?	–	✓	?
Wide bicondylar/bigonial breadth	✓	✓	?	✓	✓	?

✓ presence of a trait; **–** absence of a trait; ? trait could not be evaluated due to damage.

**Figure 6:**
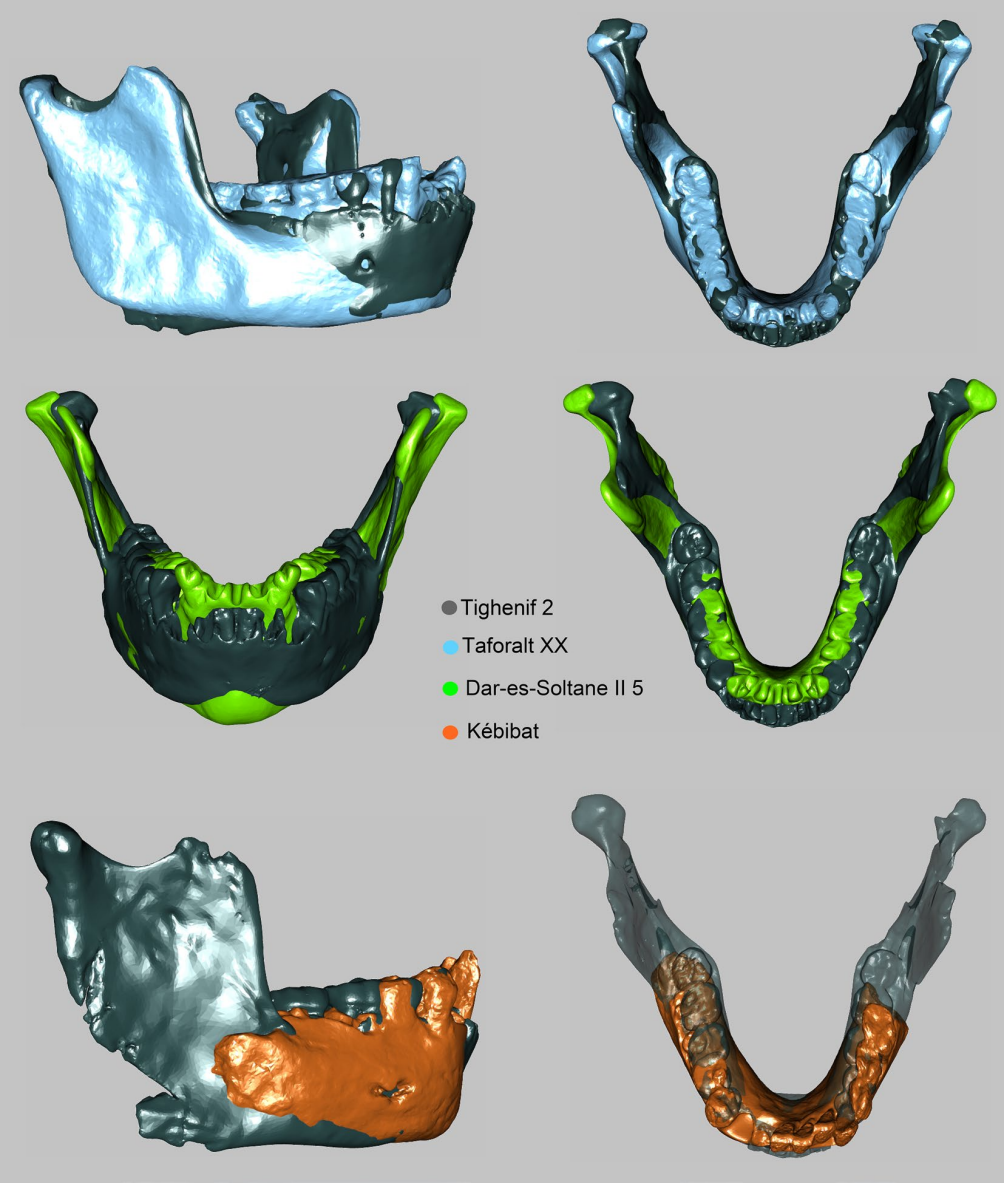
Superimpositions in Procrustes space of the reconstructed Tighenif 2 (dark gray), Kébibat (orange), the reconstructed Dar-es-Soltane II 5 (green), and Taforalt XX (blue).

**Figure 7:**
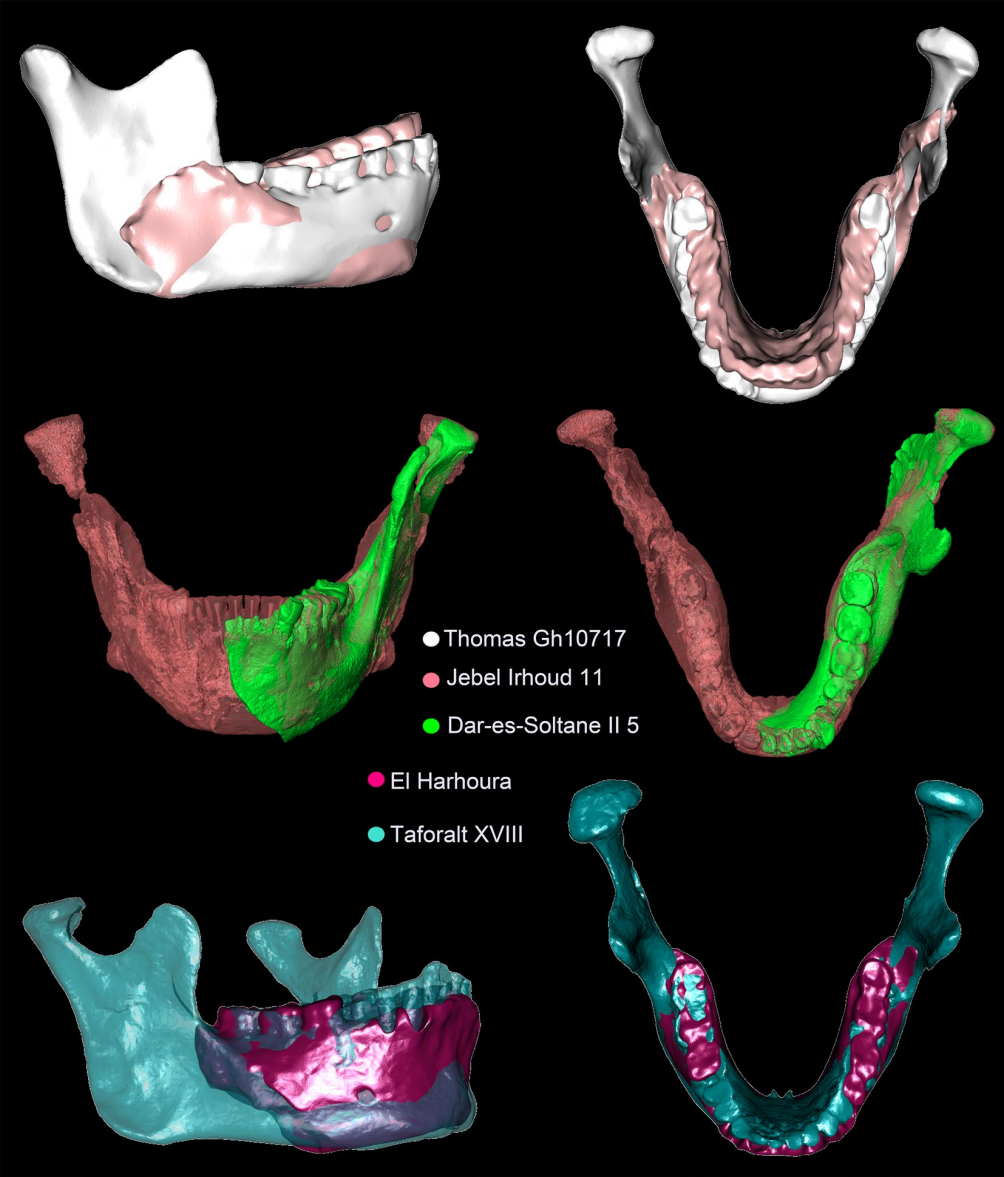
Superimpositions in Procrustes space of the original Irhoud 11 (pink), Thomas I Gh10717 (white), Dar-es-Soltane II 5 (green), El Harhoura (violet), Taforalt XVIII (turquoise), and a reconstructed version of Irhoud 11 (pink transparent).

Procrustes nearest neighbors of Maghrebian late Early/Middle Pleistocene mandibles (Tighenif, Thomas Quarry) disclose shape affinities to Iberomaurusians and recent humans from the region (Supplementary Table S2). They also reveal a close relatedness between Tighenif 2, Irhoud 11 and Dar-es-Soltane II 5 as well as between Irhoud 11 and Thomas Gh10717 – visualized by three-dimensional superimpositions (Figs. 6 and 7). These results indicate morphological continuity in the Maghreb, substantiated by numerous archaic traits that late Early/Middle Pleistocene individuals (Tighenif, Thomas Quarry, Kébibat) share with Jebel Irhoud 11 and Aterians (Table 1). One third of the closest Procrustes neighbors to Dar-es-Soltane II 5 are Neanderthals (Table S2), reflecting PCA results (Fig. 3a). According to these lines of evidence, Dar-es-Soltane II 5 is more similar in mandibular shape to Neanderthals than to Iberomaurusians. Procrustes nearest neighbors (Supplementary Table S2) and a 3D superimposition of El Harhoura versus Taforalt XVIII (Fig. 7) partially support a morphologic link between Aterian and Iberomaurusian crania^24,26^. Iberomaurusian mandibles were distinct from penecontemporaneous sub-Saharan specimens, namely Ishango, Jebel Sahaba, Mumbwa 3, Olduvai 1, Gobero, Asselar, and Shum Laka (Fig. 3a).

Kébibat has been recently classified as a phylogenetic link between North African archaic and modern populations, exhibiting diagnostic *H. sapiens* traits on the cranium, but lacking them in the mandible^8^*.* We also identified the proposed high robusticity of Kébibat, its anteroposteriorly uniform corpus height, vertical symphysis and lack of chin. Saban^27^ disclosed a considerable number of archaic characteristics that Kébibat shares with the much older mandibular fragments from Sidi Abderrahman (400 ka BP^60^) and the younger dated Contrebandiers 1 mandible, supported by our data (Table 1, Supplementary Table S2). Our PCA (Fig. 3b) substantiates the view that the “Rabat-Kébibat Man” joins a complex transition from archaic to modern populations in Africa, previously indicated by its cranial morphology^27,28^. As many diagnostic parts of the mandible are missing (retromolar area, gonion, mandibular notch) and others are damaged (symphysis, dental arcade), we could not conclusively assign Kébibat to either early *H. sapiens* or an archaic Middle Pleistocene form. The mix between archaic and modern traits persists throughout the Aterian sample and is consistent with a mosaic evolution of the *H. sapiens* lineage^24,26,27^. At the same time there is a discernable shift in the frequency of modern traits, visible as differences between individuals (Table 1, Fig. 3) and likely due to an accretion of modern traits through time. This gradual accretion develops non-linearly through time with modern characters carried by individuals dated earlier, archaic characters carried by more recent specimens^3,28^, and a variable mix of features among penecontemporaneous specimens. For example, despite a large temporal distance between them and the presence of archaic traits, Irhoud 11 and Dar-es-Soltane II 5 exhibit a strong anteroposterior height decrease in their mandibular corpus (Figs. 1 and 7). In contrast, the younger dated fossil from El Harhoura 1 completely lacks this distinct modern feature and even displays an inferior transverse torus; at the same time it has a full human chin. Such mixed morphology is corroborated by patterns of dental morphology that Aterians share with early *H. sapiens* from the Levant^23^, North^3^ and East Africa^61^. In particular, megadontia expressed in the development of mass-additive traits on the teeth^7,23^ and of large dental root dimensions^3,62^ are reminiscent of archaic hominins whereas dental tissue proportions and root shape fall already into the range of modern human variation.

From a regional perspective, resemblances in mandibular shape (Supplementary Table S2, Figs. 6 and 7) and discrete features (Table 1) indicate that the Tighenif, Thomas Quarry and Kébibat hominins were part of the same evolving lineage as the Jebel Irhoud humans, Aterians, Iberomaurusians and recent North Africans. Absolute sizes of Aterian mandibles are in the range of early *H. sapiens* and Iberomaurusians (Fig. 5). Even though we have no proof of an in-situ population succession, Aterian morphology fits the human fossil gap between Jebel Irhoud 11 and Iberomaurusians, suggesting a greater time depth for regional continuity in Northern Africa than previously established^57,58^. The archaeological hiatus at the Middle/Later Stone Age transition^29,30^ might result from a demographic bottleneck, but not from a population replacement of Aterians by Iberomaurusians. Yet, a morphological link of Dar-es-Soltane II 5 to the Iberomaurusians, as suggested by Ferembach^24^, remains vague as this group is only distantly related in mandibular shape (Table S2, Fig. 3). This concurs with a facial analysis of Dar-es-Soltane II 5^41^, yet we agree with the authors that it might also be obscured by the large size of the specimen.

A previous allometric study on human mandibles had revealed that some aspects of shape variation in adults correlate with mandibular size of the individual^11^. In this context, most Neanderthal-like shape features in early *H. sapiens* could be attributed to their large size. Interestingly, two large Aterian specimens in the current study carry similar features, namely a prognathic U-shaped dental arcade (El Harhoura), and a wide ramus with a large gonial angle as well as a wide bicondylar breadth (Dar-es-Soltane II 5). As Aterians chronologically align more with early *H. sapiens* than with later groups of humans, we assume similar allometric constraints . The amounts of shape variance explained by mandibular size in early *H. sapiens* (10.2%) and the pooled sample (4.6%) are alike^11^ (Table 2). This suggests that the Neanderthal-like appearance of Dar-es-Soltane II 5 (Fig. 3a; Table S2) might be linked to its large mandibular size (Supplementary Table S5). Bicondylar distance in Dar-es-Soltane II 5 even exceeds Neanderthal/European Middle Pleistocene means (Supplementary Tables S6, S8). Cranial and facial morphology are close to the Irhoud humans^24,41^, a sample that also shows size-driven Neanderthal-like morphology^11^. Likewise, the prognathic and U-shaped El Harhoura corpus (Table 1, Fig. 1) correlates with an exceptional size (Fig. 5).

**Table 2:** Shape variance explained by size (R^2^) in the mandible data set.

	European MP	Neanderthals	African MP	Early *H. sapiens*	Sub-Saharans	Iberomaurusian	Natufian	Upper Paleolithic	Holocene	Pooled
R^2^	0.071	0.078	0.119	0.102	0.083	0.081	0.051	0.120	0.052	0.046
p	0.99	0.43	0.81	0.67	0.094	0.24	0.38	0.055	0.01	< 0.001

*MP* Middle Pleistocene.

In principle, the presence of diagnostic *H. sapiens* features (anteroposteriorly decreasing corpus height, incipient chin) throughout the Aterian sample allows us to group it within a single evolving lineage. Aterians fill a temporal gap in the global human fossil record between early and later *H. sapiens*, their heterogenous mandibular shape demonstrates an accretional pattern for the emergence of modern morphology. Apart from regional continuity, Aterians and other ancient North Africans (Tighenif, Thomas Quarry I) resemble Natufians, sub-Saharans and Upper Paleolithic people (Supplementary Table S2), shedding some light on the nature of potential exchanges between North Africa and adjacent areas. Genetically, this finding parallels a close relatedness of Iberomaurusians to Natufians, southern Europeans^57^, and—on a smaller scale—to sub-Saharan Africans^59^. To that end, late glacial back-to-Africa migrations via the Mediterranean^63–67^ and via the Near-East^59,68^ offer explanatory scenarios. Such population movements depended either on low sea-levels during glacial periods^54,69^ or on the periodic emergence of green corridors throughout the Sahara, Sinai, Negev and Nafud deserts^70–73^. The latter also allowed for human exchanges between North Africa and the Sahelian zone, accounting for the exceptional skeletal variation and/or signs of gene flow^74,75^ in most finds from the African humid period (Later Stone Age until mid-Holocene). Among our samples, especially the El Harhoura mandible matches substantially to the Jebel Sahaba series (Supplementary Table S2).

## Conclusion

African population dynamics were determined by shifting ecological boundaries between the Mediterranean and the tropical zone^71^. During green windows of reduced aridity migration corridors through Northern Africa, the Levant and the Arabian Peninsula caused pulses of hominin/human dispersals that ultimately were followed by population contractions when humid phases came to an end^71–73^. The Sahara formed a major part of this ecotone, periodically entailing bidirectional encounters not only with the Near-East, but also with sub-Saharan Africa. In this context, Northern Africa occupies a unique position for understanding supra-regional activities of hominin groups in the Middle and Late Pleistocene. Our data substantiate sporadic human exchanges between Northern Africa, the Near-East^57,59^, Europe^57,63,64^ and sub-Saharan Africa^59^ that would have been limited to periods of favorable climatic conditions^70–73^. Nevertheless, fossil mandibles dating to the Maghrebian MSA (Kébibat, Irhoud 11, Aterian) exhibit notable similarities in shape and discrete traits to preceding and succeeding populations in the area (Table 1, Supplementary Table S2; Figs. 6 and 7), implying a long-term succession of hominins in Northern Africa. In this debate, Kébibat and the Aterians fill not only a chronological but also a morphological gap of considerable interest between the earliest currently known *H. sapiens* from Jebel Irhoud and later *H. sapiens*. At the same time, their mosaic mandibular morphology sheds some light onto Late Pleistocene diversity and is in line with an accretion of modern traits through time, consistent with an ongoing masticatory gracilization^11^.

## Supplementary Information

Supplementary Information.
