# Supplementary material for: The relevance of late MSA mandibles on the emergence of modern morphology in Northern Africa
Source: Sci Rep. 2022 May 25;12:8841. doi: 10.1038/s41598-022-12607-5 (PMC9133045; doi:10.1038/s41598-022-12607-5)
Supplement: Supplementary file 1 — Supplementary Information. [file 41598_2022_12607_MOESM1_ESM.pdf]

Supplementary Material:

The relevance of late MSA mandibles on the emergence of modern morphology in Northern Africa

Inga Bergmann[a]*, Jean-Jacques Hublin[b,a], Abdelouahed Ben-Ncer[c,] Fatima Zohra Sbihi-Alaoui[c], Philipp Gunz[a], Sarah E. Freidline[d,a]

a – Department of Human Evolution, Max Planck Institute for Evolutionary Anthropology, Deutscher Platz 6, 04103 Leipzig, Germany

b – Chaire de Paléoanthropologie, Collège de France, 11, place Marcellin Berthelot, 75005 Paris, France

c – Institut National des Sciences de l'Archéologie et du Patrimoine, Hay Riad 5, 10000 Rabat, Morocco

d – Department of Anthropology, University of Central Florida, 4000 Central Florida Blvd., Orlando, USA

*Corresponding author.

E-mail address: inga_bergmann@eva.mpg.de (I. Bergmann).

Supplementary S1

Material

All specimens used in our analyses are listed in Supplementary Table S1, with their provenience, taxonomic and temporal affiliation, reconstruction method of the 3D surface model as well as in which analyses they were included (mandible or corpus data set, see methods section). The oldest group consists of African (n = 7) and European (n = 8) Middle Pleistocene non-Neanderthals. The Neanderthal sample (*n* = 27) is European and Levantine. The early *H. sapiens* sample comprises 10 individuals from the Middle and early Late Pleistocene of Africa and Israel. The Aterian sample contains Dar-es-Soltane II H5, Contrebandiers 1 and El Harhoura **(Fig. 1)**. To complement the late MSA period in Northern Africa, we included the isolated mandible from Kébibat (**Fig. 1**). We assigned 22 mandibles to the European Upper Paleolithic (UP), 22 specimens to the Natufian, and 20 individuals to the sub-Saharan group. The Holocene group comprises 54 mandibles, covering early and mid-Holocene phases (n=20) as well as recent time periods (n=34).

Supplementary S2

Methods

Data acquisition 3D surface models were generated from computed tomography using Avizo 7.1 (FEI Visualization Sciences Group, Hillsboro), NextEngine laser scans, and photogrammetry (SOM Table S1). These imaging techniques are equivalent in accuracy and precision, imposing only minor deviations on geometric morphometric data, which are in the range of acceptable error in osteometry [1–4]. In a few instances, research quality casts were used instead of the original specimen (SOM Table S1).

Data estimation We mirror-imaged some specimens manually in Geomagic Studio 2014 v. 3.0 (Geomagic Inc., Rockhill), following the procedures described in Bergmann et al. [4]. Qafzeh 25 had been reconstructed and mirror-imaged version by Schuh et al. [5]. The hemimandible DeS II H5 was mirrored in Avizo 7.1 using an anisometric affine registration correlation with Irhoud 11 as a reference. Both specimens are similar in their dental proportions and curvature of the arcade. A co-registration by an iterative optimization algorithm suggested the location of the mirroring plane in the alveole of I1, indicating that DeS5 might have had only three incisors. The resultant mirrored mandible aligned with the preserved cranial parts of DeS5. The lack of a 4th incisor might not be uncommon in this site as also the adolescent individual DeS II H4 seems not to provide enough space for four incisors. In DeS5, setting the mirroring plane at the usual spot between the central

incisors would result in an unnaturally wide mandible, exceeding the dimensions of even Middle Pleistocene specimens.

In cases of bilaterally missing data, those were estimated in R by a thin-plate-spline interpolation that deforms the sample mean onto the deficient configuration.

Data processing We measured 31 landmarks of type I and II in a 3D coordinate system [6] at traditional anatomical points [7–9]. They are connected by 270 curve semilandmarks, capturing the most important edges and curvatures (Supplementary Fig. 1; Table 3). All semilandmarks were slid along their respective curves in order to find the position that produces the least bending energy of a thin-plate-spline deformation between each configuration and the sample mean shape [10,11]. After symmetrizing the slid data through reflected relabeling [12], they were superimposed by generalized Procrustes analyses [13]. This procedure scales all specimens to unit centroid size, thereby accounting for isometric size differences. The resultant Procrustes shape coordinates were analyzed by software routines written in R, mainly using the packages 'Morpho' v. 2.7 [14] and 'Geomorph' v. 3.3.1 [15]. To account for missing data, we created a separate data set that captures only the mandibular corpus (27 landmarks, 118 semilandmarks; **Supplementary Fig. 2**).

Data analysis Mandibular shape analysis was conducted using 3D geometric morphometric methods, an approach that controls for differences in scale. We performed principal components analyses (PCA) in shape space to explore differences between groups. Group mean shapes were calculated from Procrustes shape coordinates and warped onto a group-specific reference surface by thin-plate-spline interpolation. Likewise, we illustrated shape changes along PC1, PC2 and PC3 by warping positive and negative extremes ± 3 SD of each PC onto a reference surface. We regressed Procrustes shape coordinates on the natural log of centroid size (ln CS) to assess the amount of shape variance explained by size as R^2 value.

Supplementary Fig. 1. Landmarks (orange), semilandmarks (green), metric distances (red).

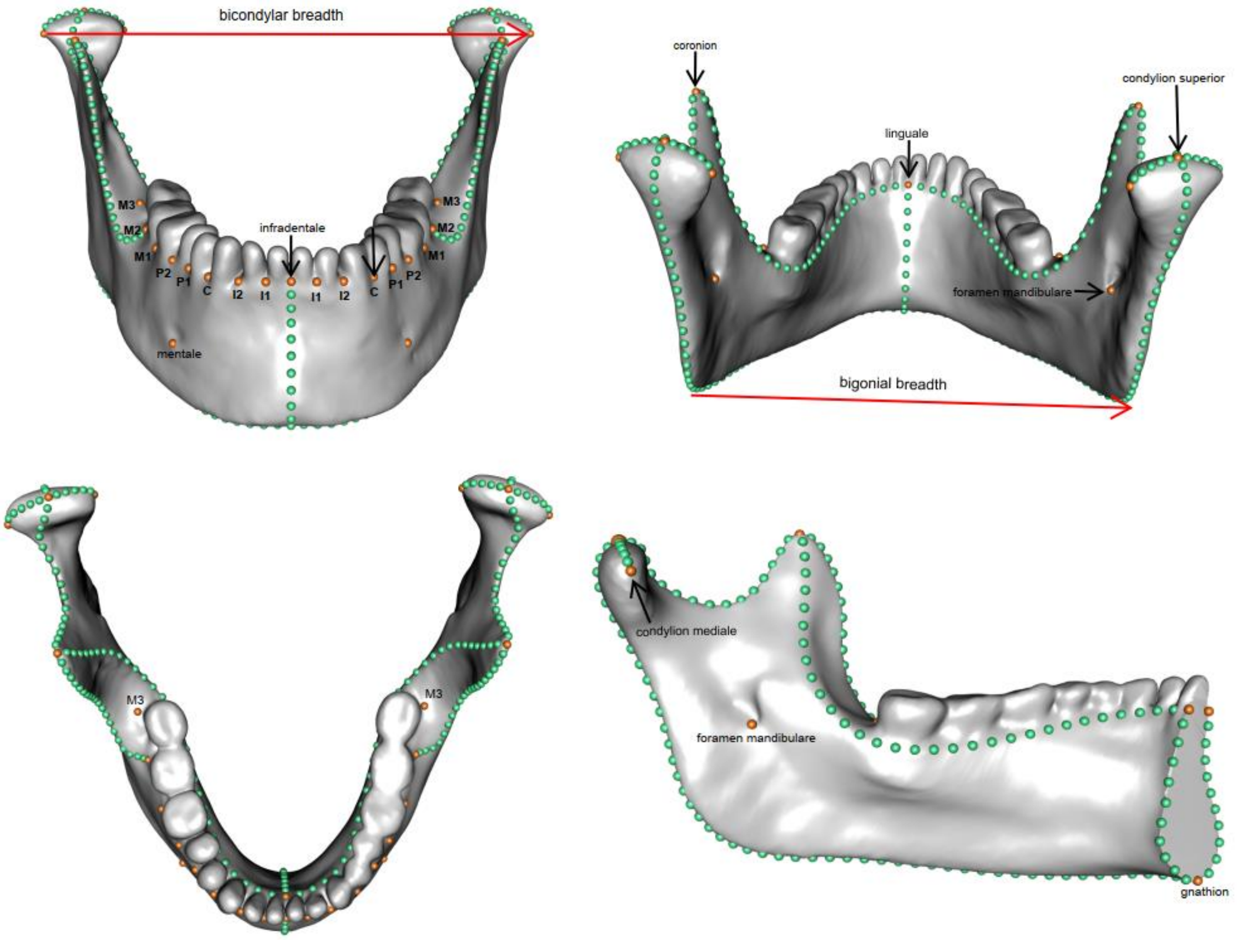

Supplementary Fig. 2. Landmarks (orange) and semilandmarks (green) in the corpus data set.

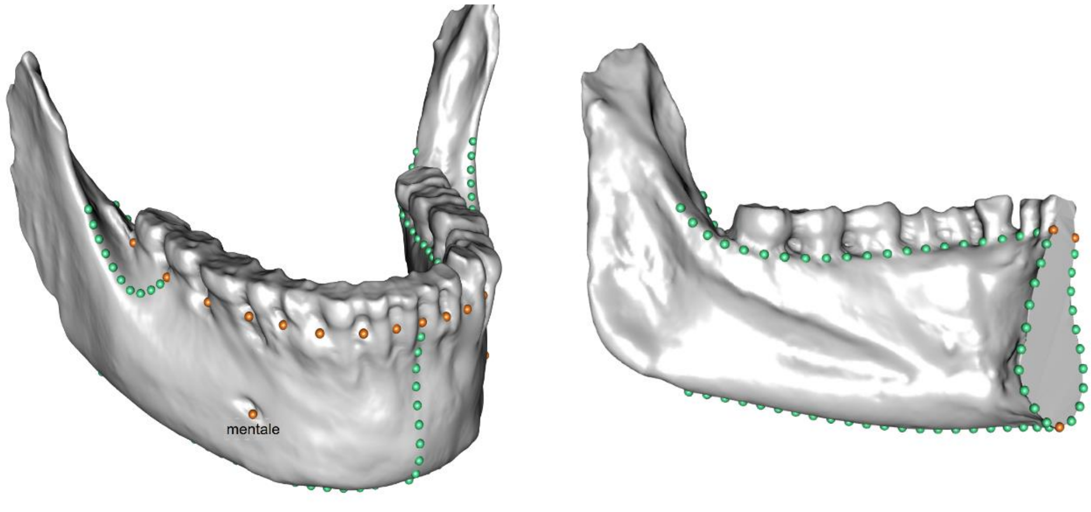

Supplementary Table 1

Specimens analyzed in this study[a].

Specimen	Sex	Origin	Dating	References	Data	Group	Scan
Arago II<br>Arago XIII[b]	F<br>M	France	500-400 ka	Crégut [16]; Yokoyama et al. [17–19]; Guérin and Faure [20]; Falguères et al. [21]	mandible	European Middle Pleistocene	PS
Mauer	M?	Germany	600 ka	Wagner et al. [22,23]	mandible	European Middle Pleistocene	CT
Montmaurin	F?	France	250-200 ka	Crégut-Bonnoure et al. [24]; Vialet et al. [25]	mandible	European Middle Pleistocene	PS
AT 250/AT 793 (individual IV)<br>AT 888/721/776 (ind. XXI/cran. V)[c]<br>AT 950 (individual XXVIII)<br>AT 952/AT 505/AT 604 (ind. XIX)	F<br>M<br>F<br>F	Spain	min. 430 ka	Arnold et al. [26]; Arsuaga et al. [27]	corpus<br>mandible<br>mandible<br>mandible	European Middle Pleistocene	PS
Baringo Kapthurin 67 (KNM-BK)<br>Baringo Kapthurin 8518 (KNM-BK)	--	Kenya	510 ka	Deino and McBrearty [28]	mandible	African Middle Pleistocene	CT
Olduvai 22[b]	--	Tanzania	Early or Mid. Pleist.	Rightmire [29,30]	corpus	African Middle Pleistocene	CT/PS
Thomas Quarry I Gh10717	--	Morocco	700-600 ka	Raynal et al. [31,32]; Geraads [33]; Rhodes et al. [34]	mandible	African Middle Pleistocene	CT
Tighenif 1 + 3<br>Tighenif 2[b]	M?<br>F?	Algeria<br>Algeria	1 Ma<br>1 Ma	Sahnouni and van der Made [35]; Geraads [36]; Martini and Geraads [37]; Geraads [38]	mandible	African Middle Pleistocene	CT/PS
Kébibat	--	Morocco	140 ka	Stearns and Thurber [39]; [40]	corpus	--------------------------------	PS
Dar-es-Soltane II H5	M?	Morocco	≤ 85-75 ka	Raynal and Occietti [41]	mandible	Aterian	CT
El Harhoura/ Grotte Zouhrah	--	Morocco	66 ka	Gallois [42]; Debénath [43]	corpus	Aterian	CT
Contrebandiers 1	M?	Morocco	100 ka	Roche and Texier [44]; Jacobs et al. [45]	mandible	Aterian	CT
Border Cave 2<br>Border Cave 5	F?<br>M?	South Africa	150 ka?<br>75 ka	Wood [46]<br>Grün et al. [47]	corpus<br>mandible	early *H. sapiens*<br>early *H. sapiens*	PS<br>PS
Djebel Irhoud 11	--	Morocco	300 ka	Richter et al. [48]	mandible	early *H. sapiens*	CT
Klasies River Mouth 13400[b]<br>Klasies River Mouth 41815	M?	South Africa	115 ka	Wood [46]	corpus<br>mandible	early *H. sapiens*<br>early *H. sapiens*	PS<br>PS
Qafzeh 9[b] + 25[b]	--	Israel	90 ka?	Wood [46]	mandible	early *H. sapiens*	CT
Skhul IV [b] + V	--	Israel	115 ka	Grün et al. [49]	mandible	early *H. sapiens*	CT
Tabun 2 C2[b]	F	Israel	122 ka	Grün and Stringer [50]	mandible	early *H. sapiens*	CT
Tabun 1[b]	--		155 ka?	Mercier et al. [51]	mandible	Neanderthal	PS

Amud 1	M	Israel	50 ka	Rink et al. [52]	mandible	Neanderthal	CT
Arcy II/Arcy 8 (Gr. d. l'Hyène)	M	France	125 ka	Leroi-Gourhan [53]; Higham et al. [54]	corpus	Neanderthal	CT
Bourgeois-Delaunay 1 (La Chaise)	--	France	125 ka	Condemi [55]	mandible	Neanderthal	CT
El Sidrón 1 + 2	M	Spain	40 ka	Wood [56]; Wood et al. [57]	mandible	Neanderthal	CT
Guattari 2	M?	Italy	55 ka	Schwarcz [58]	corpus	Neanderthal	CT
Guattari 3	M	Italy	65 ka	Grün and Stringer [59]	mandible	Neanderthal	CT
Kebara 2	M	Israel	60 ka	Schwarcz et al. [60]; Bar-Yosef et al. [61]	corpus	Neanderthal	CT
Krapina 59	--	Croatia	130 ka?	Rink et al. [62]; Williams [63]	mandible	Neanderthal	CT
Krapina 54[b]+ 55+ 57 + 58	--	Croatia	130 ka?		corpus	Neanderthal	CT
La Ferrassie 1	M	France	70 ka	Wood [46]	mandible	Neanderthal	CT
La Naulette 1	F	Belgium	Mid. Pleist.	Toussaint et al. [64]	corpus	Neanderthal	CT
La Quina 9[b]	--	France	45 ka	Debénath and Jelinek [65]	mandible	Neanderthal	PS
Le Regourdou 1	--	France	70 ka	Wood [46]	mandible	Neanderthal	CT
Saint-Césaire	--	France	35 ka	Mercier et al. [66]	mandible	Neanderthal	CT
Sima de las Palomas 6[b] +23[b]	--	Spain	45 ka?	Walker et al. [67–69]	corpus	Neanderthal	CT/PS
Spy I/ Spy 3	--	Belgium	36 ka	Semal et al. [70]	corpus	Neanderthal	CT
Vindija 11.40	--	Croatia	45 ka	Devièse et al. [71]	mandible	Neanderthal	CT
Vindija 11.39 + 11.45	--	Croatia	45 ka		corpus	Neanderthal	CT
Ehringsdorf F1009	F?	Germany	230 ka	Schüler [72]; Street et al. [73]	corpus	Neanderthal	CT
Zafarraya 2[c]	--	Spain	38 ka	Michel et al. [74]	mandible	Neanderthal	CT
Abri Pataud 1	F	France	18 ka	Chiotti et al. [75]	mandible	Upper Paleolithic	PS
Arene Candide 2	M	Italy	12.3 ka	Formicola et al. [76]	mandible	Upper Paleolithic	CT
Cro-Magnon 1 + 3	M	France	28 ka	Henry-Gambier [77]	corpus	Upper Paleolithic	PS/CT
Dolní Věstonice 3<br>Dolní Věstonice 13<br>Dolní Věstonice 14<br>Dolní Věstonice 15<br>Dolní Věstonice 16	F<br>M<br>M<br>F<br>M	Czech Republic	26.5 ka	Klima [78]; Svoboda [79]; Trinkaus and Svoboda [80]	mandible	Upper Paleolithic	CT
El Mirón 1	F	Spain	18.5 ka	Carretero et al. [81]	mandible	Upper Paleolithic	CT

Grimaldi: Barma Grande 2[b]<br>Barma Grande 4<br>Barma Grande 5[b]	M<br>F<br>M	Italy	24.5 ka	Formicola [82]	corpus<br>mandible<br>mandible	Upper Paleolithic	CT
Grotte des Enfants 4	M	Italy	25 ka	Formicola [82]; Formicola et al. [76]	mandible	Upper Paleolithic	LS
Isturitz 106<br>Isturitz 115	--<br>--	France	18.5 ka<br>18 ka	Henry-Gambier [83]	corpus<br>mandible	Upper Paleolithic	PS
Oase 1	M	Romania	40.5 ka	Trinkaus et al. [84]	mandible	Upper Paleolithic	CT
Oberkassel 1<br>Oberkassel 2	M<br>F	Germany	12 ka	Baales and Street [85]	mandible	Upper Paleolithic	CT
Sungir 1	M	Russia	33 ka	Nalawade-Chavan [86]; Trinkaus [87]	mandible	Upper Paleolithic	CT
Pavlov 1-5	M	CZ	25.5 ka	Trinkaus and Svoboda [80]	mandible	Upper Paleolithic	CT
Villabruna 1	M	Italy	14 ka	Vercellotti et al. [88]	mandible	Upper Paleolithic	CT
Afalou 28	M	Algeria	18 ka	Hachi et al. [89]; Hadjouis [90]	mandible	Iberomaurusian	PS
Afalou (*n* = 7, Iberomaurusian)	1 F<br>6M	Algeria	13 ka	Hachi et al. [89]; Hadjouis [90]	mandible	Iberomaurusian	PS
Taforalt (*n* = 9, Iberomaurusian)	7M<br>2 F	Morocco	14.5 ka	Bouzouggar et al. [91]; Barton et al. [92]	mandible	Iberomaurusian	PS
Asselar	M?	Mali	8 ka	Vialet et al. [93]	mandible	sub-Saharan	PS
Gobero1 B11<br>Gobero1 B19<br>Gobero3 B8	M<br>--<br>M	Niger	6900<br>7200-4500<br>9500	Sereno [94]	mandible	sub-Saharan	CT
Ishango 15	--	Congo	23 ka	Brooks et al. [95]; Crevecoeur et al. [96]	mandible	sub-Saharan	CT
Jebel Sahaba (*n* = 11, LP Nubian)	7M<br>4 F	Sudan	15-12 ka	[97,98]	mandible	sub-Saharan	LS
Lothagam KNM-LT13702	--	Kenya	7.5 ka	Schepartz [99]	mandible	sub-Saharan	CT
Mumbwa 3	--	Zambia	18 ka	Protsch [100]	mandible	sub-Saharan	PS
Olduvai 1	M	Tanzania	17 ka	Protsch [101]	mandible	sub-Saharan	PS
Shum Laka 6/SE III	F	Cameroun	3370-3030	Lipson et al. [102]	mandible	sub-Saharan	PS
Ain Mallaha (*n* = 9, Late Natufian)	--	Israel	10.5 ka	Valla [103]	mandible	Natufian	PS
Hayonim 17 (Early Natufian)<br>Hayonim 19 + 20 + 25 + 27<br>Hayonim 8 + 29 (Late Natufian)<br>Hayonim 29a	M<br>M<br>M<br>F?	Israel	12 ka<br>12 ka<br>11 ka<br>11 ka	Bar-Yosef and Goren [104]; Hopf and Bar-Yosef [105];<br>Belfer-Cohen [106]; Grosman [107]	corpus<br>mandible<br>mandible<br>mandible	Natufian	CT<br>CT/PS<br>CT/PS<br>PS

Nahal Oren 6 + 14 (Late Natufian)<br>Nahal Oren 8<br>Nahal Oren 18<br>Nahal Oren 20	--<br>M<br>F<br>M?	Israel	13-11 ka	Nadel and Rosenberg [108]; Grosman [107]	mandible	Natufian	PS/CT<br>CT<br>PS<br>PS
Coimbra (*n* = 13)	6M<br>7 F	Portugal	recent	Ferreira et al. [109]	mandible	Holocene	LS
Grotte de Gramat (*n* = 2)	M	France	7 ka	Valdeyron et al. [110]	mandible	Holocene	PS
Indian mandible	--	India	recent	J.-J. Hublin's unpublished data	mandible	Holocene	PS
Schela Cladovei (*n* = 6)	M	Romania	8 ka	pers. comm. Ekaterina Stansfield (York)	mandible	Holocene	CT
ULAC 13<br>ULAC 66<br>ULAC 258 + 259<br>ULAC 336 + 337 +703<br>ULAC 498 + 522 + 536 + 566 + 607<br>ULAC 753 + 759 + 760 + 762<br>ULAC 767<br>ULAC 769<br>ULAC 770<br>ULAC 779	F<br>F<br>M<br>F<br>M<br>M<br>M<br>M<br>F<br>M	Germany<br>Scandin.<br>Algeria<br>Egypt<br>Egypt<br>Guinea<br>Congo<br>W-Africa<br>W-Africa<br>S-Africa	recent	Schmidt [111]	mandible	Holocene	CT
Vărăşti Gulmeniţa (*n* = 12)	M	Romania	5.5 ka	pers. comm. Ekaterina Stansfield (York)	mandible	Holocene	CT

Abbreviations: PS = photogrammetry scan; LS = laser scan; CT = computed tomography; F = female; M = male.

[a] Sex estimation as reported in the cited literature. The series from Afalou and Klasies River, and the individual Gramat 2 were sex-estimated by the author, considering traditional traits of the mandible and cranium. Coimbra was a known-sex series.

[b] One side has been mirror-imaged, for details see the methods section.

[c] Cast.

Supplementary Table S2. Procrustes Nearest Neighbors[a] of Thomas Gh10717, Tighenif 1-3, Jebel Irhoud 11, Kébibat[b], Dar-es-Soltane II 5, Contrebandiers 1, El Harhoura[b]

Thomas	Tighenif 1	Tighenif 2	Tighenif 3	Irhoud 11	Kébibat	Dar-es-Soltane 5	Contrebandiers 1	El Harhoura
sub-Saharan	Jebel Sahaba	**Tighenif 3**	**Tighenif 2**	Jebel Sahaba	Neanderthal	Neanderthal	Natufian	Jebel Sahaba
European MP	Natufian	African MP	**recent Egyptian**	Natufian	**Contrebandiers 1**	Neanderthal	sub-Saharan	Jebel Sahaba
sub-Saharan	Upper Paleolithic	Natufian	recent W-Africa	Upper Paleolithic	Jebel Sahaba	Upper Paleolithic	**Iberomaurusian**	**Iberomaurusian**
recent Guinea	European MP	**Iberomaurusian**	**Iberomaurusian**	Natufian	Upper Paleolithic	Neanderthal	sub-Saharan	Jebel Sahaba
early *H. sapiens*	Jebel Sahaba	**Iberomaurusian**	Holocene	**Iberomaurusian**	**Iberomaurusian**	**Jebel Irhoud 11**	Upper Paleolithic	Jebel Sahaba
Upper Paleolithic	sub-Saharan	European MP	recent Portuguese	Upper Paleolithic	Natufian	Natufian	European MP	European MP
Upper Paleolithic	recent Guinean	Natufian	**recent Egyptian**	Neanderthal	Natufian	early *H. sapiens*	Upper Paleolithic	Jebel Sahaba
Upper Paleolithic	Natufian	Natufian	Neandertha	**Iberomaurusian**	Jebel Sahaba	European MP	early *H. sapiens*	Jebel Sahaba
**Jebel Irhoud 11**	early *H. sapiens*	Neanderthal	**Iberomaurusian**	Upper Paleolithic	**Iberomaurusian**	Upper Paleolithic	Upper Paleolithic	**Iberomaurusian**
**Iberomaurusian**	early *H. sapiens*	**recent Egyptian**	**recent Egyptian**	Holocene	recent W-Africa	European MP	Natufian	Jebel Sahaba
Upper Paleolithic	Upper Paleolithic	recent Portuguese	Holocene	sub-Saharan	Neanderthal	Natufian	recent Portuguese	recent Portuguese
Jebel Sahaba	Jebel Sahaba	Upper Paleolithic	**recent Egyptian**	Natufian	early *H. sapiens*	European MP	Natufian	sub-Saharan
Upper Paleolithic	Upper Paleolithic	Upper Paleolithic	recent Portuguese	Natufian	**Iberomaurusian**	**Tighenif 2**	early *H. sapiens*	Natufian
African MP	Neanderthal	Neanderthal	**Iberomaurusian**	European MP	Upper Paleolithic	sub-Saharan	Upper Paleolithic	sub-Saharan
Sub-Saharan	**recent Algerian**	**Dar-es-Soltane II 5**	Holocene	Natufian	Upper Paleolithic	Natufian	**recent Egyptian**	early *H. sapiens*
European MP	sub-Saharan	Holocene	**Iberomaurusian**	**Thomas Gh10717**	Jebel Sahaba	European MP	**Iberomaurusian**	Natufian
early *H. sapiens*	Jebel Sahaba	**recent Egyptian**	Natufian	Neanderthal	**El Harhoura**	Upper Paleolithic	recent Portuguese	recent Guinean
recent South African	sub-Saharan	recent Guinean	recent Guinean	early *H. sapiens*	sub-Saharan	Neanderthal	recent German	Natufian
Natufian	recent W-Africa	**Jebel Irhoud 11**	recent Egyptian	sub-Saharan	Neanderthal	Natufian	Natufian	Neanderthal
Natufian	sub-Saharan	Natufian	recent W-Africa	**Dar-es-Soltane II 5**	European MP	Neanderthal	**Iberomaurusian**	**Iberomaurusian**
sub-Saharan	Jebel Sahaba	early *H. sapiens*	recent Congo	Jebel Sahaba	Natufian	**Iberomaurusian**	recent Portuguese	Neanderthal
Jebel Sahaba	recent South African	Natufian	**recent Algerian**	Jebel Sahaba	recent Guinean	Neanderthal	European MP	Natufian
**Iberomaurusian**	**Thomas Gh10717**	sub-Saharan	Natufian	Natufian	Jebel Sahaba	early *H. sapiens*	Holocene	Holocene
Jebel Sahaba	Upper Paleolithic	**recent Algerian**	Natufian	Neanderthal	Natufian	recent Egyptian	European MP	Jebel Sahaba

Abbreviations: MP = Middle Pleistocene

[a] Northern African individuals in bold [b] computed in the corpus data set

Supplementary Table S3

Centroid size (CS) per group in the mandible data set (*n* = 166), ranked by size.

Group	CS mean
African Middle Pleistocene	1048
Neanderthals	1032
European Middle Pleistocene	1031
Early *H. sapiens*	1008
Iberomaurusian	986
sub-Saharan	962
Upper Paleolithic	945
Natufian	912
Holocene	912

Supplementary Table S4

Centroid size (CS) per group of the corpus data set (*n* = 192), ranked by size.

Group	CS mean
African Middle Pleistocene	457
European Middle Pleistocene	435
Early *H. sapiens*	434
Iberomaurusian	434
Neanderthals	430
sub-Saharan	423
Upper Paleolithic	418
Natufian	407
Holocene	402

Supplementary Table S5

Centroid sizes of Jebel Irhoud, North African Middle Pleistocene and Aterian samples in both data sets.

Specimen	CS mandible	CS corpus
Tighenif 1	1200	508
Tighenif 2	982	434
Tighenif 3	1221	527
Thomas Quarry 10717	885	391
Jebel Irhoud 11	1078	465
Kébibat	---	381
Contrebandiers 1	976	436
Dar-es-Soltane II 5	1127	468
El Harhoura 1	---	483

Supplementary Table S6

Bicondylar breadth in mm per group (*n* = 166).

Group	Bicondylar breadth
Neanderthals	140
European Middle Pleistocene	139
African Middle Pleistocene	130
Early *H. sapiens*	130
Iberomaurusian	124
Upper Paleolithic	122
sub-Saharan	119
Natufian	118
Holocene	115

Supplementary Table S7

Bigonial breadth in mm per group (*n* = 166).

Group	Bigonial breadth
Iberomaurusian	105
Neanderthals	99
Upper Paleolithic	98
Early *H. sapiens*	97
African Middle Pleistocene	96
sub-Saharan	95
European Middle Pleistocene	93
Holocene	92
Natufian	90

Supplementary Table S8

Bicondylar and bigonial breadth of North African samples. All figures in mm.

Specimen	Bicondylar breadth	Bigonial breadth
Tighenif 1	139	113
Tighenif 2	125	91
Tighenif 3	159	114
Thomas Quarry 10717	107	90
Jebel Irhoud 11	146	103
Dar-es-Soltane II 5	159	99
Contrebandiers 1	132	101

Supplementary Figure S3. Principal component analysis of the pooled mandible data set (n = 166) in shape space, showing PC1 vs. PC2. Percentages show the amount of variance accounted for.

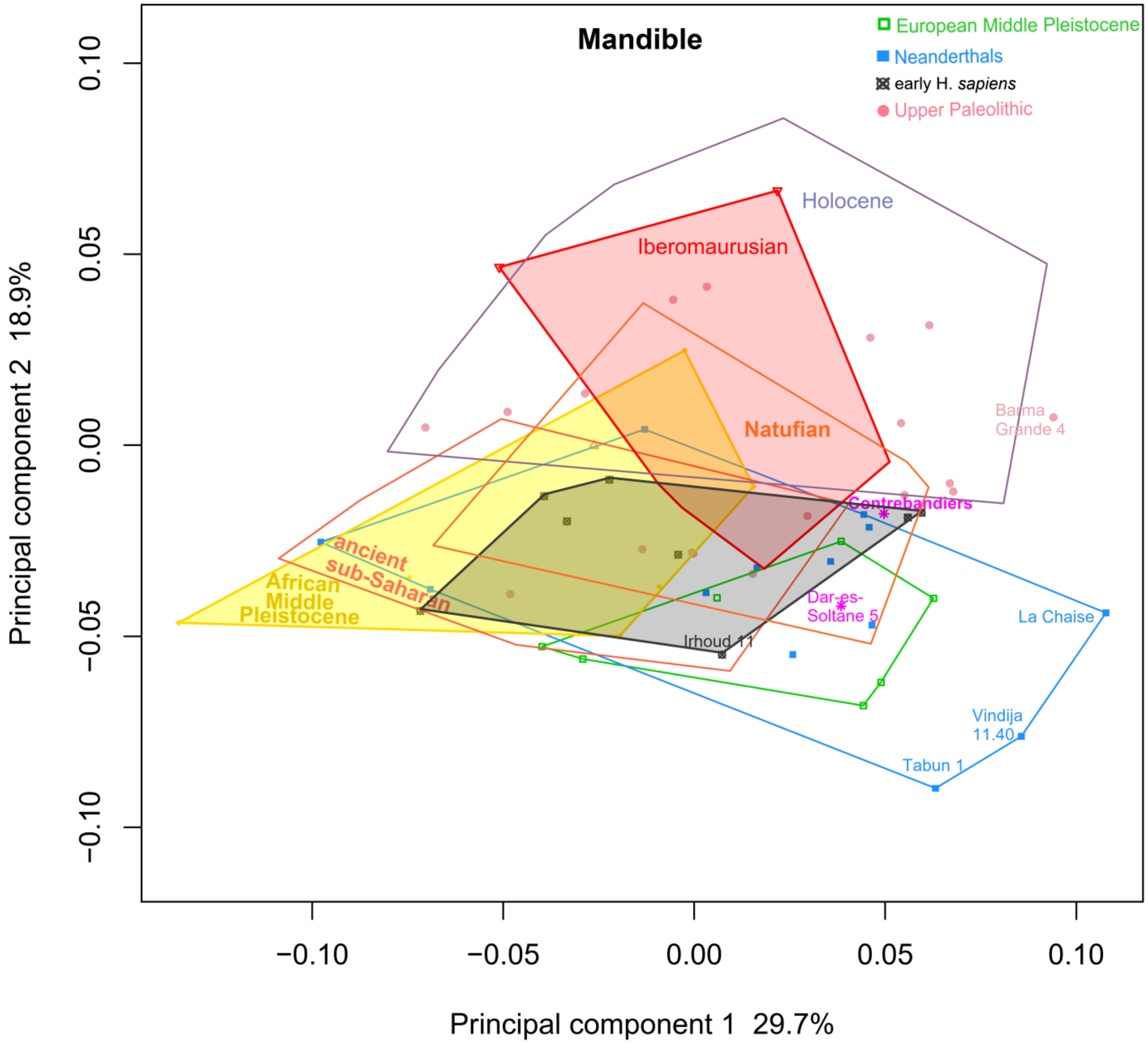

Supplementary References

1. Katz, D. & Friess, M. Technical note: 3D from standard digital photography of human crania—a preliminary assessment. *Am. J. Phys. Anthropol.* **154**, 152–158 (2014).
2. Jurda, M. & Urbanová, P. Three-dimensional documentation of Dolní Věstonice skeletal remains: can photogrammetry substitute laser scanning? *Anthropol. 1962-* **54**, 109–118 (2016).
3. Buzi, C. *et al.* Measuring the shape: performance evaluation of a photogrammetry improvement applied to the Neanderthal skull Saccopastore 1. *Acta Imeko* **7**, 79–85 (2018).
4. Bergmann, I., Hublin, J.-J., Gunz, P. & Freidline, S. E. How did modern morphology evolve in the human mandible? The relationship between static adult allometry and mandibular variability in Homo sapiens. *J. Hum. Evol.* **157**, 103026 (2021).
5. Schuh, A. *et al.* La mandibule de l'adulte Qafzeh 25 (Paléolithique Moyen, Basse Galilée). Reconstruction virtuelle 3D et analyse morphométrique. *Paléorient* **43**, 49–59 (2016).
6. Bookstein, F. L. Introduction to methods for landmark data. in *Proceedings of the Michigan morphometrics workshop* vol. 2 215–226 (The University of Michigan Museum of Zoology Special Publication Number 2, 1990).
7. von Török, A. Über Variationen und Korrelationen der Neigungsverhältnisse am Unterkiefer. *Zs F Ethnol.* **30**, 125–182 (1898).
8. Martin, R. & Saller, K. *Lehrbuch der Anthropologie in systematischer Darstellung. Fischer Verlag, Stuttgart. - Google-Suche*. (Fischer Verlag, 1956).
9. Bräuer, G. Anthropometrie. in *Antropologie. Handbuch der vergleichenden Biologie des Menschen I* 129–232 (Fischer Verlag, 1988).
10. Gunz, P., Mitteroecker, P. & Bookstein, F. L. Semilandmarks in three dimensions. in *Modern morphometrics in physical anthropology* (ed. Slice, D.) 73–98 (Springer, 2005).
11. Gunz, P. & Mitteroecker, P. Semilandmarks: a method for quantifying curves and surfaces. *Hystrix Ital. J. Mammal.* **24**, 103–109 (2013).
12. Mardia, K. V., Bookstein, F. L. & Moreton, I. J. Statistical assessment of bilateral symmetry of shapes. *Biometrika* **87**, 285–300 (2000).

13. Rohlf, F. J. & Slice, D. Extensions of the Procrustes method for the optimal superimposition of landmarks. *Syst. Biol.* **39**, 40–59 (1990).

14. Schlager, S. Morpho and Rvcg – Shape analysis in R: R-Packages for geometric morphometrics, shape analysis and surface manipulations. in *Statistical Shape and Deformation Analysis* (eds. Zheng, G., Li, S. & Székely, G.) 217–256 (Academic Press, 2017).

15. Adams, D., Collyer, M. & Kaliontzopoulou, A. Geometric Morphometric Analyses of 2D/3D Landmark Data. (2019).

16. Crégut, E. La faune de mammifères du gisement pléistocène moyen antérissien de la Caune de l'Arago (Tautavel, Pyrénées-Orientales, France). *Comptes Rendus L'Académie Sci. Paris* **290**, 751–754 (1980).

17. Yokoyama, Y., Van Nguyen, H. & Rapp, P. J. Datation directe de l'Homme de Tautavel par la spectrométrie gamma, non destructive, du crâne humain fossile Arago XXI. *Comptes Rendus L'Académie Sci. Paris* **292**, 741–744 (1981).

18. Yokoyama, Y. *et al.* ESR dating of stalagmites of the Caune de l'Arago, the Grotte de Lazaret, the Grotte du Vallonnet and the Abri Pie Lombard: a comparison with the U-Th method. in *3rd specialist seminar on TL and ESR dating* (1983).

19. Yokoyama, Y., Falgueres, C. & Quaegebeur, J. P. ESR dating of quartz from quaternary sediments: First attempt. *Nucl. Tracks Radiat. Meas. 1982* **10**, 921–928 (1985).

20. Guérin, C. & Faure, M. Biochronologie et datations absolues : convergence de méthodes dans différents sites du Paléolithique ancien. *ArchéoSciences Rev. Archéom.* **15**, 41–46 (1991).

21. Falguères, C. *et al.* New U-series dates at the Caune de l'Arago, France. *J. Archaeol. Sci.* **31**, 941–952 (2004).

22. Wagner, G. A. *et al.* Radiometric dating of the type-site for Homo heidelbergensis at Mauer, Germany. *Proc. Natl. Acad. Sci.* **107**, 19726–19730 (2010).

23. Wagner, G. A., Maul, L. C., Löscher, M. & Schreiber, H. D. Mauer – the type site of Homo heidelbergensis: palaeoenvironment and age. *Quat. Sci. Rev.* **30**, 1464–1473 (2011).

24. Crégut-Bonnoure, É. *et al.* Le contexte géomorphologique et faunique de l'homme de Montmaurin (Haute-Garonne). *Préhistoires Méditerranéennes* (2010).

25. Vialet, A. *et al.* Re-examination of “ancient” fossils discovered in the middle of the 20th century. Late Middle Pleistocene hominins from Montmaurin caves (Southwest of France). *J. Int. Union Prehist. Protohistoric Sci.* **2**, 32–37 (2019).

26. Arnold, L. J. *et al.* Luminescence dating and palaeomagnetic age constraint on hominins from Sima de los Huesos, Atapuerca, Spain. *J. Hum. Evol.* **67**, 85–107 (2014).

27. Arsuaga, J. L. *et al.* Neandertal roots: Cranial and chronological evidence from Sima de los Huesos. *Science* **344**, 1358–1363 (2014).

28. Deino, A. L. & McBrearty, S. 40 Ar/39 Ar dating of the Kapthurin Formation, Baringo, Kenya. *J. Hum. Evol.* **42**, 185–210 (2002).

29. Rightmire, G. P. Middle Pleistocene hominids from Olduvai Gorge, Northern Tanzania. *Am. J. Phys. Anthropol.* **53**, 225–241 (1980).

30. Rightmire, G. P. Tautavel hominids and Homo erectus from Olduvai Gorge. *Congrès Int. Paléontol. Hum. 1er Congrès* 798–813 (1982).

31. Raynal, J.-P. *et al.* Hominid Cave at Thomas Quarry I (Casablanca, Morocco): Recent findings and their context. *Quat. Int.* **223–224**, 369–382 (2010).

32. Raynal, J. P., Sbihi Alaoui, F. Z., Geraads, D., Magoga, L. & Mohi, A. The earliest occupation of North-Africa: the Moroccan perspective. *Quat. Int.* **75**, 65–75 (2001).

33. Geraads, D. Plio-Pleistocene Mammalian biostratigraphy of Atlantic Morocco. in *Quaternaire* vol. 13 43–53 (2002).

34. Rhodes, E. J., Singarayer, J. S., Raynal, J.-P., Westaway, K. E. & Sbihi-Alaoui, F. Z. New age estimates for the Palaeolithic assemblages and Pleistocene succession of Casablanca, Morocco. *Quat. Sci. Rev.* **25**, 2569–2585 (2006).

35. Sahnouni, M. & van der Made, J. The Oldowan in North Africa within a biochronological framework. in *The cutting edge: new approaches to the archaeology of human origins* 179–210 (Stone Age Institute Press Bloomington, 2009).

36. Geraads, D. Pleistocene Carnivora (Mammalia) from Tighennif (Ternifine), Algeria. *Geobios* **49**, 445–458 (2016).

37. Martini, P. & Geraads, D. Camelus thomasi Pomel, 1893 from the Pleistocene type-locality Tighennif (Algeria). Comparisons with modern Camelus. *Geodiversitas* **40**, 115–134 (2018).
38. Geraads, D. Tighennif (Ternifine), Algeria: Environments of the earliest human fossils of North Africa in the late Early/early Middle Pleistocene. in *African Paleoecology and Human Evolution* (Cambridge University Press, In press).
39. Stearns, C. E. & Thurber, D. L. Th230/U234 dates of late Pleistocene marine fossils from the Mediterranean and Moroccan littorals. *Prog. Oceanogr.* **4**, 293–305 (1965).
40. Chahid, D. *et al.* New lithostratigraphic and chronostratigraphic data for the fossil human skull-bearing eolianite of Rabat-Kebibat (Morocco). *Quat. Rev. Assoc. Fr. Pour Létude Quat.* 249–261 (2020) doi:10.4000/quaternaire.14287.
41. Raynal, J.-P. & Occhietti, S. Amino Chronology and an Earlier Age for the Moroccan Aterian. in *Modern Origins: A North African Perspective* (eds. Hublin, J.-J. & McPherron, S. P.) 79–90 (Springer Netherlands, 2012). doi:10.1007/978-94-007-2929-2_6.
42. Gallois, B. Thermoluminescence et interactions de couplage dans des cristaux dopés avec des ions terres rares: application à la mise au point d'une nouvelle méthode en chronologie absolue: la gamma-thermoluminescence. (Université de Bordeaux 1, 1980).
43. Debénath, A. Le peuplement préhistorique du Maroc: données récentes et problèmes. *L'Anthropologie* **104**, 131–145 (2000).
44. Roche, J. & Texier, J.-P. Découverte de restes humains dans un niveau atérien supérieur de la grotte des Contrebandiers, à Temara (Maroc). *Comptes Rendus L'Académie Sci. Paris Sér. D* **282**, 45–47 (1976).
45. Jacobs, Z. *et al.* Single-grain OSL dating at La Grotte des Contrebandiers ('Smugglers' Cave'), Morocco: improved age constraints for the Middle Paleolithic levels. *J. Archaeol. Sci.* **38**, 3631–3643 (2011).
46. Wood, B. *Wiley-Blackwell Encyclopedia of Human Evolution*. (John Wiley & Sons, 2011).
47. Grün, R., Beaumont, P., Tobias, P. V. & Eggins, S. On the age of Border Cave 5 human mandible. *J. Hum. Evol.* **45**, 155–167 (2003).

48. Richter, D. *et al.* The age of the hominin fossils from Jebel Irhoud, Morocco, and the origins of the Middle Stone Age. *Nature* **546**, 293–296 (2017).

49. Grün, R. *et al.* U-series and ESR analyses of bones and teeth relating to the human burials from Skhul. *J. Hum. Evol.* **49**, 316–334 (2005).

50. Grün, R. & Stringer, C. Tabun revisited: revised ESR chronology and new ESR and U-series analyses of dental material from Tabun C1. *J. Hum. Evol.* **39**, 601–612 (2000).

51. Mercier, N. *et al.* TL Dates of Burnt Flints from Jelinek's Excavations at Tabun and their Implications. *J. Archaeol. Sci.* **22**, 495–509 (1995).

52. Rink, W. J. *et al.* Electron spin resonance (ESR) and thermal ionization mass spectrometric (TIMS) 230Th/234U dating of teeth in Middle Paleolithic layers at Amud Cave, Israel. *Geoarchaeology* **16**, 701–717 (2001).

53. Leroi-Gourhan, A. Étude des restes humains fossiles provenant des grottes d'Arcy-sur-Cure. *Ann. Paléontol.* **44**, 87–148 (1958).

54. Higham, T. *et al.* Chronology of the Grotte du Renne (France) and implications for the context of ornaments and human remains within the Châtelperronian. *Proc. Natl. Acad. Sci. U. S. A.* **107**, 20234–20239 (2010).

55. Condemi, S. *Les Néandertaliens de La Chaise: Abri Bourgeois-Delaunay*. vol. 15 (Comité des travaux historiques et scientifiques-CTHS, 2001).

56. Wood, R. E. A new date for the Neanderthals from El Sidrón Cave (Asturias, northern Spain). *Archaeometry* **55**, 148–158 (2013).

57. Wood, R. E. *et al.* Radiocarbon dating casts doubt on the late chronology of the Middle to Upper Palaeolithic transition in southern Iberia. *Proc. Natl. Acad. Sci.* **110**, 2781–2786 (2013).

58. Schwarcz, H. P. On the reexamination of Grotta Guattari: uranium-series and electron-spin-resonance dates. *Curr. Anthropol.* **32**, 313–316 (1991).

59. Grün, R. & Stringer, C. B. Electron spin resonance dating and the evolution of modern humans. *Archaeometry* **33**, 153–199 (1991).

60. Schwarcz, H. P. *et al.* ESR dating of the Neanderthal site, Kebara Cave, Israel. *J. Archaeol. Sci.* **16**, 653–659 (1989).

61. Bar-Yosef, O. *et al.* The Dating of the Upper Paleolithic Layers in Kebara Cave, Mt Carmel. *J. Archaeol. Sci.* **23**, 297–306 (1996).
62. Rink, W. J., Schwarcz, H. P., Smith, F. H., & others. ESR ages for Krapina hominids. *Nature* **378**, 24–24 (1995).
63. Williams, F. L. A comparison of the Krapina lower facial remains to an ontogenetic series of Neandertal fossils. *Period. Biol.* **108**, 279–288 (2006).
64. Toussaint, M., Semal, P. & Pirson, S. Les Néandertaliens du bassin mosan belge: bilan 2006-2011. in *Le Paléolithique moyen en Belgique. Mélanges Marguerite Ulrix-Closset* (eds. Toussaint, M., Di Modica, K. & Pirson, S.) vol. 128 105–148 (Service de Préhistoire, 2011).
65. Debénath, A. & Jelinek, A. J. Nouvelles fouilles à La Quina (Charente): résultats préliminaires. *Gall. Préhistoire* **40**, 29–74 (1998).
66. Mercier, N. *et al.* Thermoluminescence dating of the late Neanderthal remains from Saint-Césaire. *Nature* **351**, 737–739 (1991).
67. Walker, M. J. *et al.* Late Neandertals in Southeastern Iberia: Sima de las Palomas del Cabezo Gordo, Murcia, Spain. *Proc. Natl. Acad. Sci.* **105**, 20631–20636 (2008).
68. Walker, M. J., Lombardi, A. V., Zapata, J. & Trinkaus, E. Neandertal mandibles from the Sima de las Palomas del Cabezo Gordo, Murcia, southeastern Spain. *Am. J. Phys. Anthropol.* **142**, 261–272 (2010).
69. Walker, M. J. *et al.* The excavation of buried articulated Neanderthal skeletons at Sima de las Palomas (Murcia, SE Spain). *Quat. Int.* **259**, 7–21 (2012).
70. Semal, P. *et al.* New data on the late Neandertals: Direct dating of the Belgian Spy fossils. *Am. J. Phys. Anthropol.* **138**, 421–428 (2009).
71. Devièse, T. *et al.* Direct dating of Neanderthal remains from the site of Vindija Cave and implications for the Middle to Upper Paleolithic transition. *Proc. Natl. Acad. Sci.* **114**, 10606–10611 (2017).
72. Schüler, T. ESR-Datierung von Zahnschmelz aus dem Unteren Travertin von Weimar-Ehringsdorf. *Alt-Thüring.* **28**, 9–23 (1994).
73. Street, M., Terberger, T. & Orschiedt, J. A critical review of the German Paleolithic hominin record. *J. Hum. Evol.* **51**, 551–579 (2006).

74. Michel, V., Delanghe-Sabatier, D., Bard, E. & Ruiz, C. B. U-series, ESR and 14 C studies of the fossil remains from the Mousterian levels of Zafarraya Cave (Spain): A revised chronology of Neandertal presence. *Quat. Geochronol.* **15**, 20–33 (2013).

75. Chiotti, L., Nespoulet, R. & Henry-Gambier, D. Occupations and status of the Abri Pataud (Dordogne, France) during the Final Gravettian. *Quat. Int.* **359–360**, 406–422 (2015).

76. Formicola, V., Pettitt, P. B., Maggi, R. & Hedges, R. Tempo and mode of formation of the Late Epigravettian necropolis of Arene Candide cave (Italy): direct radiocarbon evidence. *J. Archaeol. Sci.* **32**, 1598–1602 (2005).

77. Henry-Gambier, D. Les fossiles de Cro-Magnon (Les Eyzies-de-Tayac, Dordogne) : nouvelles données sur leur position chronologique et leur attribution culturelle. *PALEO Rev. Archéologie Préhistorique* **14**, 201–204 (2002).

78. Klima, B. A triple burial from the Upper Paleolithic of Dolní Věstonice, Czechoslovakia. *J. Hum. Evol.* **16**, 831–835 (1988).

79. Svoboda, J. A new male burial from Dolní Věstonice. *J. Hum. Evol.* **16**, 827–830 (1988).

80. Trinkaus, E. & Svoboda, J. *Early Modern Human Evolution in Central Europe: the People of Dolní Věstonice and Pavlov*. vol. 12 (Oxford University Press, 2006).

81. Carretero, J. M. *et al.* The Magdalenian human remains from El Mirón Cave, Cantabria (Spain). *J. Archaeol. Sci.* **60**, 10–27 (2015).

82. Formicola, V. A direct AMS radiocarbon date on the Barma Grande 6 Upper Paleolithic skeleton. *Identity Temporality Moral Geogr.* **45**, 114–118 (2004).

83. Henry-Gambier, Dominique. Datation radiocarbone directe et attribution culturelle des vestiges humain paléolithiques de la grotte d'Isturitz (Pyrénées-Atlantiques). *Bulletin de la Société préhistorique française* vol. 110 645–656 (2013).

84. Trinkaus, E. *et al.* An early modern human from the Peştera cu Oase, Romania. *Proc. Natl. Acad. Sci.* **100**, 11231–11236 (2003).

85. Baales, M. & Street, M. Late Palaeolithic backed-point assemblages in the northern Rhineland: current research and changing views. *Notae Praehistoricae* **18**, 77–92 (1998).

86. Nalawade-Chavan, S., McCullagh, J. & Hedges, R. New Hydroxyproline Radiocarbon Dates from Sungir, Russia, Confirm Early Mid Upper Palaeolithic Burials in Eurasia. *PLoS ONE* **9**, (2014).

87. Trinkaus, E., Buzhilova, A. P., Mednikova, M. B. & Dobrovolskaya, M. V. The age of the Sunghir Upper Paleolithic human burials. *Anthropol. 1962-* **53**, 221–231 (2015).

88. Vercellotti, G., Alciati, G., Richards, M. P. & Formicola, V. The Late Upper Paleolithic skeleton Villabruna 1 (Italy): a source of data on biology and behavior of a 14.000 year-old hunter. *J Anthr. Sci* **86**, 143–163 (2008).

89. Hachi, S. *et al.* Figurines du Paléolithique supérieur en matière minérale plastique cuite d'Afalou Bou Rhummel (Babors, Algérie). Premières analyses par spectroscopie d'absorption Infrarouge. *L'Anthropologie* **106**, 57–97 (2002).

90. Hadjouis, D. Les hommes du Paléolithique supérieur d'Afalou Bou Rhummel (Bedjaia, Algérie). Interprétation nouvelle des cinétiques cranio-faciales et des effets de l'avulsion dentaire. Malformations crâniennes, troubles de la croissance, anomalies et maladies alvéolo-dentaires. *L'Anthropologie* **106**, 337–375 (2002).

91. Bouzouggar, A. *et al.* Reevaluating the Age of the Iberomaurusian in Morocco. *Afr. Archaeol. Rev.* **25**, 3–19 (2008).

92. Barton, R. N. E. *et al.* Origins of the Iberomaurusian in NW Africa: New AMS radiocarbon dating of the Middle and Later Stone Age deposits at Taforalt Cave, Morocco. *J. Hum. Evol.* **65**, 266–281 (2013).

93. Vialet, A., André, L. & Aoudia, L. L'Homme fossile d'Asselar (actuel Mali). Étude critique, mise en perspective historique et nouvelles interprétations. *L'Anthropologie* **117**, 345–361 (2013).

94. Sereno, P. C. *et al.* Lakeside Cemeteries in the Sahara: 5000 Years of Holocene Population and Environmental Change. *PLoS ONE* **3**, (2008).

95. Brooks, A. S. *et al.* Dating and context of three middle stone age sites with bone points in the Upper Semliki Valley, Zaire. *Science* **268**, 548–553 (1995).

96. Crevecoeur, I., Brooks, A., Ribot, I., Cornelissen, E. & Semal, P. Late Stone Age human remains from Ishango (Democratic Republic of Congo): New insights on Late Pleistocene modern human diversity in Africa. *J. Hum. Evol.* **96**, 35–57 (2016).

97. Zazzo, A. Bone and enamel carbonate diagenesis: A radiocarbon prospective. *Palaeogeogr. Palaeoclimatol. Palaeoecol.* **416**, 168–178 (2014).

98. Usai, D. The Qadan, the Jebel Sahaba Cemetery and the Lithic Collection. *Archaeol. Pol.* **58**, 99–119 (2020).

99. Schepartz, L. A. From hunters to herders: subsistence pattern and morphological change in eastern Africa. (University of Michigan, 1987).

100. Protsch, R. R. R. Mumbwa: Its absolute chronology and archaeology. *Z. Für Morphol. Anthropol.* **68**, 1–7 (1977).

101. Protsch, R. The age and stratigraphic position of Olduvai Hominid I. *J. Hum. Evol.* **3**, 379–385 (1974).

102. Lipson, M. *et al.* Ancient West African foragers in the context of African population history. *Nature* **577**, 665–670 (2020).

103. Valla, F. Raymond. Les fouilles de Ain Mallaha (Eynan) de 2003 à 2005 : quatrième rapport préliminaire. *Mitekufat Haeven* **37**, 135–379 (2007).

104. Bar-Yosef, O. & Goren, N. Natufian remains in Hayonim cave. *Paléorient* **1**, 49–68 (1973).

105. Hopf, M. & Bar-Yosef, O. Plant remains from Hayonim cave, western Galilee. *Paléorient* **13**, 117–120 (1987).

106. Belfer-Cohen, A. The Natufian graveyard in Hayonim cave. *Paléorient* **14**, 297–308 (1988).

107. Grosman, L. The Natufian chronological scheme-New insights and their implications. in *Natufian foragers in the Levant: terminal Pleistocene social changes in Western Asia* vol. 19 622–637 (2013).

108. Nadel, D. & Rosenberg, D. Late Natufian Nahal Oren and its satellite sites: some regional & ceremonial aspects. *Farming* **2011**, 1–16 (2011).

109. Ferreira, M. T. *et al.* A new forensic collection housed at the University of Coimbra, Portugal: The 21st century identified skeletal collection. *Forensic Sci. Int.* **245**, 202.e1–5 (2014).

110. Valdeyron, N. *et al.* Le gisement du Cuzoul de Gramat (Lot, France): présentation des nouveaux travaux et résultats préliminaires. in *Marges, Frontières et Transgressions. Actualité de la Recherche. Actes des 8e Rencontres Méridionales de Préhistoire Récente* (eds. Sénépart, I., Perrin, T., Thiraut, E. & Bonnardin, S.) 197–211 (Archives d'Écologie Préhistorique, 2011).

111. Schmidt, E. Die anthropologischen Sammlungen Deutschlands. *Leipz. Sect.* (1886).
